# *Bacillus* SEVA siblings: A Golden Gate-based toolbox to create personalized integrative vectors for *Bacillus subtilis*

**DOI:** 10.1038/s41598-017-14329-5

**Published:** 2017-10-26

**Authors:** Jara Radeck, Daniel Meyer, Nina Lautenschläger, Thorsten Mascher

**Affiliations:** 1 Institute of Microbiology, Technische Universität (TU) Dresden, 01062 Dresden, Germany; 2 Department of Biology I, Ludwig-Maximilians-Universität (LMU) München, 82152 Planegg-Martinsried, Germany

**Keywords:** Expression systems, Bacterial genes

## Abstract

*Bacillus subtilis* combines natural competence for genetic transformation with highly efficient homologous recombination. These features allow using vectors that integrate into the genome via double homologous recombination. So far, their utilization is restricted by the fixed combination of resistance markers and integration loci, as well as species- or strain-specific regions of homology. To overcome these limitations, we developed a toolbox for the creation of personalized *Bacillus* vectors in a standardized manner with a focus on fast and easy adaptation of the sequences specifying the integration loci. We based our vector toolkit on the Standard European Vector Architecture (SEVA) to allow the usage of their vector parts. The *Bacillus* SEVA siblings are assembled via efficient one-pot Golden Gate reactions from four *entry* parts with the choice of four different enzymes. The toolbox contains seven *Bacillus* resistance markers, two *Escherichia coli* origins of replication, and a free choice of integration loci. Vectors can be customized with a cargo, before or after vector assembly, and could be used in different *B. subtilis* strains and potentially beyond. Our adaptation of the SEVA-standard provides a powerful and standardized toolkit for the convenient creation of personalized *Bacillus* vectors.

## Introduction

### The diversity of plasmid vectors in molecular cloning

In molecular cloning, vectors are vehicles to transfer foreign nucleic acids into a living cell. In the context of this article, we restrict the term “vector” to “plasmid vectors”, small circular DNA molecules that originate from bacteria. They are easy to handle for inserts up to 10–15 kb, replicate independently of the bacterial chromosome and can be isolated in large amounts through standard plasmid preparation procedures. Over the last decades, vectors were increasingly modified to meet custom needs. For instance, they may contain promoters for ready-to-use gene expression, or reporter genes to measure transcription or translation rates, respectively. More elaborated vector types can contain biosafety features, internal measuring standards or may be used for clean chromosomal gene deletions or dual expression systems^1–4^. Synthetic biology strives to implement and extend the principles of engineering (standardization, decoupling, abstraction) into biology^5^. Especially standardization in vector and plasmid construction offers the clear advantage of comparability, compatibility, flexibility and reusability of single parts and whole vectors, as exemplified by the Standard European Vector Architecture (SEVA).

### The Standard European Vector Architecture provides standardized vectors for Gram-negative bacteria

In 2013, the group of Victor de Lorenzo developed a standardized vector toolbox for the use in Gram-negative bacteria, with a special interest in *Pseudomonas putida*. With SEVA, they set the stage for a community-driven development platform and for evolving a standardized vector collection. This platform facilitates finding, creating, and naming of suitable vectors as well as their downstream handling^6,7^. Currently, the SEVA database lists 135 SEVA vectors and 49 SEVA siblings^8^ (http://seva.cnb.csic.es/, March 2017). Subsequently, linker sequences were developed to make the vectors compatible to different cloning methods^9^.

**Figure 1:**
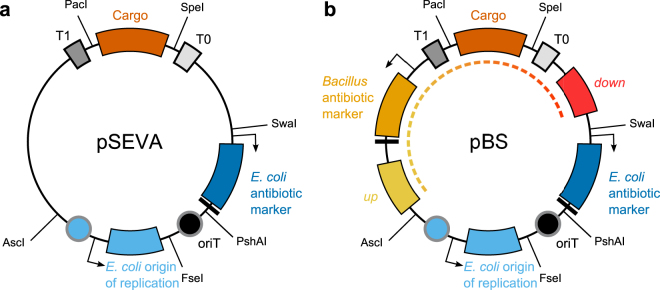
Configuration of a SEVA vector and its *Bacillus* SEVA sibling. (**a**) The basic SEVA vector is composed of six parts, separated by defined endonuclease restriction sites. The transcriptional terminators T0 and T1 as well as the origin of transfer *oriT* are fixed, whereas the cargo, the ori and the antibiotic marker can be chosen freely from a pool of SEVA-compatible parts. (**b**) For genomic integration, three parts were added to the SEVA layout to create the *Bacillus* SEVA sibling pBS: Flanking homology regions *up* and *down*, as well as an antibiotic marker for *Bacillus*. Vector verification and propagation occurs in *E. coli* and only the part in between the homology regions (dashed line) will integrate into the genome. Vectors are drawn not to scale. Functional transcriptional units are indicated with an arrow (promoter) and black bar (terminator).

Each SEVA vector contains at least three functional elements (Fig. 1a), (i) the origin of replication (ori) for vector replication in the host cell, (ii) a selectable marker, e.g. an antibiotic resistance cassette to select for vector uptake and maintenance, and (iii) a multiple cloning site (MCS) for insertion of the DNA of interest. In the SEVA standard, the latter part is called cargo, irrespective of the size and nature of the insert. The cargo is isolated by flanking double transcriptional terminators (T1, T0), to avoid unwanted transcriptional read-through into other elements of the backbone. All parts are flanked by defined rare endonuclease restriction sites and assemble in a fixed order and orientation (Fig. 1a). They must not contain these and further restriction endonuclease recognition sites. Additionally, the transcriptional terminators and the origin of transfer (*oriT*, for plasmid conjugation) are predefined, whereas the selectable marker, ori and cargo can be chosen freely, as long as they adhere to the standard. An easy number-based nomenclature assures the fast determination of vector features from the vector’s name^6,7^.

### Distinct features of vectors for *Bacillus subtilis*

*Bacillus subtilis* is the best studied low-G + C Gram-positive bacterium (phylum *Firmicutes*) and one of the leading workhorses of the biotechnological industry^10–12^. Its ease of genetic manipulation is based on its natural competence, which includes the active uptake of (any) DNA and recombination of homologous regions into the chromosome^13^. These features enable to routinely use integrative instead of replicative vectors. This ensures genetic stability even without maintaining a constant selective pressure. Moreover, copy number effects are avoided, which can otherwise influence the promoter activity on replicative vectors, particularly at the single cell level^14,15^.

For cloning convenience, vectors designed for integration into the *B. subtilis* genome usually replicate in *E. coli* but do not contain an ori for *B. subtilis*. Instead, two homology regions of at least 400 bp, which define the insertion locus, flank the cargo and the resistance marker for *B. subtilis*. The plasmids are linearized in the *E. coli* part of the vector before *B. subtilis* transformation to avoid integration via single crossing over events. Consequently, only the DNA segment that is located between the homology regions will integrate into the chromosome, resulting in genetic stability of single copy number inserts even in the absence of selective pressure.

The currently available vectors combine known and effective selection cassettes with well-characterized integration loci^16–20^. This status quo limits the combination of resistance markers and integration sites, and does not allow targeting entirely new chromosomal regions. Moreover, existing vectors cannot be used in another *Bacillus* strain, which differs in the nucleotide sequence at the specified integration site.

Due to the genetic accessibility of *B. subtilis*, PCR-based methods, such as long-flanking homology (LFH)-PCR^21^, can be used to target new or strain-specific loci. While this approach is very convenient for generating knock-out mutants, it is restricted with respect to the cargo: fusing several PCR products, and obtaining large fragments, e.g. the *luxABCDE* bioluminescence reporter operon (5.7 kb) that allows online measurement^17,19^, with sufficient yield for efficient transformation (>1 µg^22^) can be tedious. In these cases, transformants have to be sequenced each time to ensure the integrity of the cargo (e.g. lack of mutations). In order to combine the advantages of vectors (stability and reusability) with those of PCR-based methods (flexibility) for the use in *B. subtilis* and related species, we designed, constructed and tested a new vector concept: the *Bacillus* SEVA siblings.

### Concept of *Bacillus* SEVA siblings as customized vectors

Here, we describe a toolkit that was developed for the fast and easy generation of genomic integration vectors for *B. subtilis*, thereby overcoming the traditional limitations described above. Each vector contains a resistance marker, cargo and flanking homology regions of choice. To ensure compatibility and reusability of already existing parts for replication in *E. coli*, we based our system on SEVA and added flanking homology regions as well as *Bacillus* resistance markers at defined positions (Fig. 1b). Our vectors will be named *Bacillus* SEVA siblings, which is in line with the current SEVA regulations^6^.

Our toolbox offers seven functional antibiotic resistance cassettes for selection in *B. subtilis* and high and medium copy number *E. coli* vectors modified to be assembled with your homology regions of interest in a one-pot Golden Gate assembly^23,24^. We tested the assembly efficiencies for five different enzymes and the *B. subtilis* transformability of every part provided. As proof of concept for efficient assembly and functionality, we analyzed the expression levels of the red fluorescing protein mKate2 under control of the xylose-inducible promoter P_*xylA*_ at different chromosomal insertion loci.

## Results

### Vector layout for double cross-over homologous recombination in *B. subtilis*

We designed and constructed our vector building toolkit with the main goal to easily exchange the target loci for double homologous integration into the genome. This is important for two major reasons: Current vector collections do not allow the free combination of integration loci and selectable markers. Moreover, they omit the use of *Bacillus* strains if they differ from the reference lab strains in their nucleotide sequence, so that the integration sites of standard vectors are not compatible. For our collection of customizable vectors, we focus on allelic replacement via double recombination, where the DNA-sequence to be integrated into the chromosome (here termed *integration part*) is flanked by two regions of homology (Fig. 1b). For *B. subtilis*, approximately 400 bp of identical sequence are required for efficient integration. Shorter sequences (70 bp) can sometimes be sufficient, but are not recommended due to the significantly reduced efficiency^25^. For ease of construction, the *integration part* is combined with an ori and selectable marker for *E. coli* (here termed *replication part*). Before *B. subtilis* transformation, the vector is linearized in the *replication part* to avoid single cross-over events and ensure double cross-over integration.

Although our toolkit was optimized for the exchange of integration sites, it can be customized in manifold ways as will be outlined in the discussion. A quick user manual with helpful information for vector construction can be found in the supplemental material (Text S1).

### The customizable vector collection is based on SEVA to allow re-use of *E. coli* parts

Currently, most vectors and plasmids used for genetic manipulation of *B. subtilis* are propagated in *E. coli*. As demonstrated for NarK and β-carotene production, the *E. coli* vector propagation strongly depends on the ori and resistance markers in ways that cannot be foreseen yet^9^. Consequently, we designed our vectors to be flexible not only with respect to their cargo, but also their ori and *E. coli* resistance marker. For this purpose, we based our vector toolkit on SEVA. This standard was designed for vectors to allow exchanging of the cargo, resistance marker and ori, using defined rare type II restriction enzymes^7^. The accompanying vector collection was very well-received by scientists and is still growing. It can be used to adapt the vectors of our toolbox, which are therefore named *Bacillus* SEVA siblings. SEVA restriction sites were removed from critical parts of the *entry vectors*, so that the *final vectors* adhere to the SEVA standard, if no forbidden restriction sites are present in the customized parts.

### *Bacillus* SEVA siblings are assembled *de novo* from multiple fragments via Golden Gate cloning

As depicted in Fig. 1b, the *integration* and *replication parts* of an integration vector are separated by the regions of homology, called *up* and *down*. Consequently, the exchange of both *up* and *down* fragments with standard cloning techniques would involve two cloning and verification steps without a selectable marker. To avoid laborious stepwise cloning, advanced cloning techniques for the easy, fast, efficient and directed one-pot assembly of multiple fragments are available, such as Gibson assembly^26,27^ or Golden Gate cloning^23,24^. The latter is based on a ligase and type IIS endonucleases (e.g. BsaI), which cut outside (next to) their recognition site. Consequently, the restriction site can be designed according to need and separated from the recognition site during the cloning procedure. The reaction mix can include linear and circular DNA, containing restriction sites for the same enzyme but different overhangs. This allows the easy and efficient assembly of up to ten parts in the correct order and loss of the recognition sites in the *final vector*^23^. For our vector toolbox, we chose Golden Gate assembly for the following reasons: (i) Gibson assembly usually must be established in a lab to run smoothly, whereas restriction based cloning is more robust. (ii) The *up* and *down* regions need to be PCR-generated and Gibson assembly asks for longer overhangs thus increasing the primer costs.

**Figure 2:**
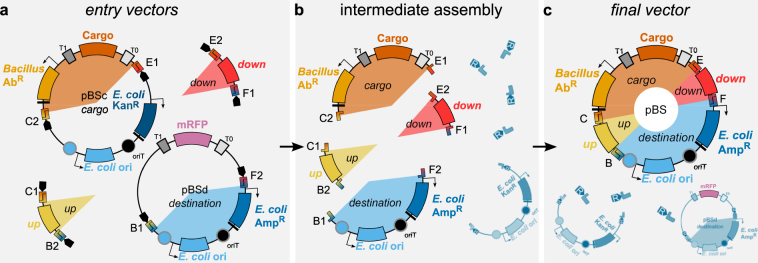
Assembly of a *Bacillus* SEVA sibling pBS. (**a**) Collection of *entry* parts needed for the assembly of a pBS vector: one *cargo vector*, one *destination vector*, one *up* and one *down* flanking homology fragment. The latter two are depicted as PCR fragments, but can also be located on a vector. Each of the desired fragments is flanked by IIS-restriction sites where the recognition site (R) is located outside the desired fragment. The compatibility of the resulting overhangs is indicated with letters and a color gradient, e.g. E1 and E2 overhangs can anneal. (**b**) Intermediate stage of the Golden Gate assembly, showing the desired fragments and some of the by-products (grey). (**c**) Creation of the *final vector*, including some possible by-products (grey). Only the *destination vector* and the *final vector* carry the ampicillin resistance marker and will be selected for after transformation of the reaction mix into *E. coli*. The *destination vector* will be counter-selected by a red/white screen based on an mRFP-marker.

Instead of re-using and modifying preexisting vectors, each new vector will be freshly assembled by combining four fragments via Golden Gate assembly (Fig. 2): the *up* and *down* fragments, the *replication part*, and the *cargo* which includes an antibiotic marker for selection in *Bacillus* and a MCS. Therefore, all vectors and sequences offered through the *Bacillus* Genetic Stock Center (BGSC) and the SEVA collection (see Table 1 and supplementary data S2) are *entry* vectors to allow customized assembly, but no *final Bacillus* vectors.

**Table 1:** Vectors of the *Bacillus* SEVA siblings toolbox.

BGSC^*^	Name^$^	Description^#^	Resistance in *E. coli* / *B. subtilis*	Source
**Vectors for default assembly**				
**Destination vectors**				
ECE701	pBSd141R	*mRFP1*, MCS-IIS F2, *bla*, ori pRO1600/ColE1, MCS-IIS B1	Amp^r^/—	This study
ECE702	pBSd191R	*mRFP1*, MCS-IIS F2, *bla*, ori pBR322/ROP, MCS-IIS B1	Amp^r^/—	This study
**Vectors for flanking homology regions** ^**§**^				
	pSEVA243	*lacZα*-pUC18 MCS, *neo*, ori pRO1600/ColE1	Kan^r^/—	SEVA^7^
ECE703	pSEVA243X	lacZα**-pUC18 MCS incl. MCS-IIS B2 + C1 for *up*, *neo*, ori pRO1600/ColE1	Kan^r^/—	This study
ECE704	pSEVA243Y	lacZα**-pUC18 MCS incl. MCS-IIS E2 + F1 for *down*, *neo*, ori pRO1600/ColE1	Kan^r^/—	This study
**Cargo-Resistance vectors**				
ECE706	pBSc241B	MCS-default, MCS-IIS E1, *neo*, ori pRO1600/ColE1, MCS-IIS C2, *bleO*	Kan^r^/ble^r^	This study
ECE707	pBSc241C	MCS-default, MCS-IIS E1, *neo*, ori pRO1600/ColE1, MCS-IIS C2, *cat*	Kan^r^/cm^r^	This study
ECE708	pBSc241M	MCS-default, MCS-IIS E1, *neo*, ori pRO1600/ColE1, MCS-IIS C2, *ermC*	Kan^r^/MLS^r^	This study
ECE709	pBSc241S	MCS-default, MCS-IIS E1, *neo*, ori pRO1600/ColE1, MCS-IIS C2, *aad(9)*	Kan^r^/spc^r^	This study
ECE710	pBSc241T	MCS-default, MCS-IIS E1, *neo*, ori pRO1600/ColE1, MCS-IIS C2, *tetL*	Kan^r^/tet^r^	This study
ECE711	pBSc241Z	MCS-default, MCS-IIS E1, *neo*, ori pRO1600/ColE1, MCS-IIS C2, *ble-Sh*	Kan^r^/zeo^r^	This study
ECE720	pBSc291K	MCS-default, MCS-IIS E1, *neo*, ori pBR322/ROP, MCS-IIS C2, *aph(3’)IIIa*	Kan^r^/kan^r^	This study
ECE713	pBSc243B	lacZα*-pUC18 MCS, MCS-IIS E1, *neo*, ori pRO1600/ColE1, MCS-IIS C2, *bleO*	Kan^r^/ble^r^	This study
ECE714	pBSc243C	lacZα*-pUC18 MCS, MCS-IIS E1, *neo*, ori pRO1600/ColE1, MCS-IIS C2, *cat*	Kan^r^/cm^r^	This study
ECE715	pBSc243M	lacZα*-pUC18 MCS, MCS-IIS E1, *neo*, ori pRO1600/ColE1, MCS-IIS C2, *ermC*	Kan^r^/MLS^r^	This study
ECE716	pBSc243S	lacZα*-pUC18 MCS, MCS-IIS E1, *neo*, ori pRO1600/ColE1, MCS-IIS C2, *aad(9)*	Kan^r^/spc^r^	This study
ECE717	pBSc243T	lacZα*-pUC18 MCS, MCS-IIS E1, *neo*, ori pRO1600/ColE1, MCS-IIS C2, *tetL*	Kan^r^/tet^r^	This study
ECE718	pBSc243Z	lacZα*-pUC18 MCS, MCS-IIS E1, *neo*, ori pRO1600/ColE1, MCS-IIS C2, *ble-Sh*	Kan^r^/zeo^r^	This study
ECE721	pBSc293K	lacZα*-pUC18 MCS, MCS-IIS E1, *neo*, ori pBR322/ROP, MCS-IIS C2, *aph(3’)IIIa*	Kan^r^/kan^r^	This study
**Vectors for customizable resistance markers**				
ECE705	pBSc241	MCS-default, MCS-IIS E1, *neo*, ori pRO1600/ColE1, MCS-IIS C2	Kan^r^/−	This study
ECE712	pBSc243	lacZα*-pUC18 MCS, MCS-IIS E1, *neo*, ori pRO1600/ColE1, MCS-IIS C2	Kan^r^/—	This study
ECE719	pBSc291	MCS-default, MCS-IIS E1, *neo*, ori pBR322/ROP, MCS-IIS C2	Kan^r^/—	This study
ECE725	pBSc293	lacZα*-pUC18 MCS, MCS-IIS E1, *neo*, ori pBR322/ROP, MCS-IIS C2	Kan^r^/—	This study
ECE722	pBSc391	MCS-default, MCS-IIS E1, *cat*, ori pBR322/ROP, MCS-IIS C2	Cm^r^/—	This study
ECE726	pBSc393	lacZα*-pUC18 MCS, MCS-IIS E1, *cat*, ori pBR322/ROP, MCS-IIS C2	Cm^r^/—	This study
**Plasmids for activity-test**				
pSEVA243X-amyE		pSEVA243X-derivative carrying a 550 bp *amyE up* fragment	Kan^r^/—	This study
pSEVA243Y-amyE		pSEVA243Y-derivative carrying a 580 bp *amyE down* fragment	Kan^r^/—	This study
pBSc241M_P_*xylA*_-*mkate2* (2195)		pBSc241M-derivative carrying *mkate2* under the control of xylose-inducible P_*xylA*_	Kan^r^/—	This study
pBS141M-amyE_mkate2		P_*xylA*_-*mkate2*,‘*amyE*, *bla*, ori pRO1600/ColE1, *amyE’*, *erm*	Amp^r^/MLS^r^	This study
pBS141M-ypqP_mkate2		P_*xylA*_-*mkate2*,‘*ypqP*, *bla*, ori pRO1600/ColE1, *ypqP’*, *erm*	Amp^r^/MLS^r^	This study
pBS141M-ykoS_mkate2		P_*xylA*_-*mkate2*,‘*ykoS*, *bla*, ori pRO1600/ColE1, *ykoS’*, *erm*	Amp^r^/MLS^r^	This study
pBS191M-ndk_mkate2		P_*xylA*_-*mkate2*,‘*ndk*, *bla*, ori pBR322/ROP, *ndk’*, *erm*	Amp^r^/MLS^r^	This study
pBS141M-thrC_mkate2		P_*xylA*_-*mkate2*,‘*thrC*, *bla*, ori pRO1600/ColE1, *thrC’*, *erm*	Amp^r^/MLS^r^	This study
**Vectors used for vector construction (templates for PCR reactions)**				
pDG148		*bla*, *kan*, *ble/phle*, P_*spac*_	Amp^r^/kan^r^	37,40
pDG780		pBluescriptKS + , *kan*	Kan^r^/kan^r^	^41^
pBS3Clux		pAH328 derivative; *sacA’…‘sacA, luxABCDE, bla, cat*	Amp^r^/cm^r^	17
pBS4S		pDG1731 derivative; *thrC’…‘thrC, ‘hom, thrB’, spc, bla*	Amp^r^/spc^r^	17
pSB1A3-mkate-B0014		*bla*, BBa_K823051 (Bsu codon-adapted red fluorescing protein *mkate2*, terminator B0014), pMB1 ori (high copy number)	Amp^r^/—	Lab stock

^*^Bacillus Genetic Stock Center (BGSC, http://www.bgsc.org/).

^$^The vector names act as identifiers for the SEVA or SEVA siblings collection. **p**, Plasmid. **BS**, *Bacillus* SEVA sibling. **d**, *destination vector*. **c**, *cargo vector.* **f**, *final vector*. Numbers according to SEVA standard: 1^st^ position, resistance marker (1, amp. 2, kan). 2^nd^ position, origin of replication (4, pRO1600/ColE1, a narrow-host-range ori with high copy number in *E. coli* and varying copy number in *Pesudomonas aeruginosa* and close relatives^7^). 9, pBR322/ROP (medium copy number ori in *E. coli* and few other bacteria^42^). 3^rd^ position, cargo (1, MCS default. 3, *lacZα*-pUC18 MCS which allows for blue-white screening with X-Gal).

^#^lacZα*, premature stop codon (C202A, Q68Stop). lacZα**, premature stop codon (pSEVA243X: G390A, W130Stop, pSEVA243Y: G399A, W133Stop). Both variants are still suitable for blue-white screening. Genes encoding antibiotic resistance markers are explained in detail in Table 3.

^§^Flanking homology regions can be stored in those vectors. If the PCR fragment contains the restriction sites needed for assembly, it can be ligated blunt end into pSEVA243 via SmaI. If restriction sites should be added for all enzymes, the PCR fragments can be ligated blunt end into pSEVA243X (for *up*) or pSEVA243Y (for *down*) via EcoRV. In this case, the correct orientation needs to be verified by sequencing.

### Architecture of *Bacillus* SEVA siblings

Figure 1 compares a *final Bacillus* SEVA **s**ibling (pBS), with a standard SEVA vector for *E. coli*. SEVA suggests a designated location for the insertion of special features that are not part of the cargo. They are positioned at the terminator sequences and next to, but not obstructing the AscI and SwaI recognition sites which allow the exchange of SEVA vector parts. In line with this regulation, the homology regions and resistance marker are located at both terminator sequences. The respective *Bacillus* resistance cassette is placed between the cargo and the T0-terminator and directed counter-clockwise in order to not interfere with the transcription of the cargo. SEVA-vectors are named according to their features in a number-based code, see SEVA 2.0 for a comprehensive description^6^. We suggest the naming of *final vectors* to be based on the SEVA-standard, in which the *E. coli* features are specified in three digits: the first digit describes the *E. coli* resistance marker, e.g. 1 for ampicillin resistance or 2 for kanamycin. The second digit indicates the ori, e.g. 4 for pRO1600/ColE1 or 9 for pBR322/ROP. The third digit encodes the cargo, e.g. 1 for the default MCS or 3 for the *lacZα*-pUC18 MCS. pSEVA243 consequently is a vector mediating kanamycin resistance [2] with a high copy number [4 = pRO1600/ColE1] that carries a MCS for blue/white screening [3 = *lacZα*-pUC18]. *Bacillus*-specific features should be added behind, so pBS143K-amyE is a vector with an *E. coli* ampicillin resistance marker [1], pRO1600/ColE1 ori [4] and *lacZα*-pUC18 MCS [3] that carries a kanamycin *Bacillus* resistance marker [K] and integrates into the *amyE*-locus [amyE].

We conceptualized the assembly so that SEVA-parts can be exchanged before or after the assembly of the *final vectors*, e.g. to accommodate the presence of either SEVA- or Golden Gate-forbidden restriction sites.

### Golden Gate shuffling to assemble *Bacillus* SEVA siblings with type IIS restriction enzymes

In our current set-up, four different fragments are needed to assemble the *final vector*: the *up* and *down* homology fragments, the *cargo* with the *Bacillus* resistance cassette and the part for replication and selection in *E. coli*, which we call *destination vector*. These *entry* parts can be combined using Golden Gate assembly as detailed in Fig. 2. The *up* and *down* homology fragments can be used either as PCR products (as depicted) or as cargo of the specialized vectors containing the *up* (pSEVA243X) and *down* (pSEVA243Y) fragments, respectively. Each entry part is flanked by recognition sites for a type IIS restriction endonuclease, which creates overhangs that allow for the directional assembly of all *entry* parts. Compatible overhangs are named with the same capital letter in Fig. 2a.

As necessary for Golden Gate assembly, the recognition sites are located “outside” of the part desired for the assembly, so that correctly assembled parts cannot be re-cleaved – in contrast to the re-ligation products of *entry* vectors. By this means, assembly of mostly correct *final vectors* is ensured. The *destination vector* carries a different antibiotic marker than all other *entry* vectors to select for vectors carrying the correct backbone. An mRFP1-cassette present on the original *destination vector* is used for red/white screening. This part is removed during assembly of the *final vector* so that colonies carrying the original vector appear red and colonies carrying the correct *final vector* appear white.

Classic Golden Gate assembly uses BsaI and BbsI (=BpiI) as type IIS restriction enzymes^23,28^, but BsmBI and BtgZI were recently found to also be suitable^29,30^. Our *Bacillus* SEVA siblings toolbox accommodates all four of them for assembly to circumvent compatibility issues with the desired genomic region, e.g. the presence of one or more recognition sites in the *up* and *down* fragments. In addition to BsaI, BbsI, BsmBI and BtgZI, we also included a fifth restriction enzyme (AarI)– not reported previously for its use in Golden Gate assembly.

### Golden Gate assembly restriction sites are arranged in special MCS-IIS

**Figure 3:**
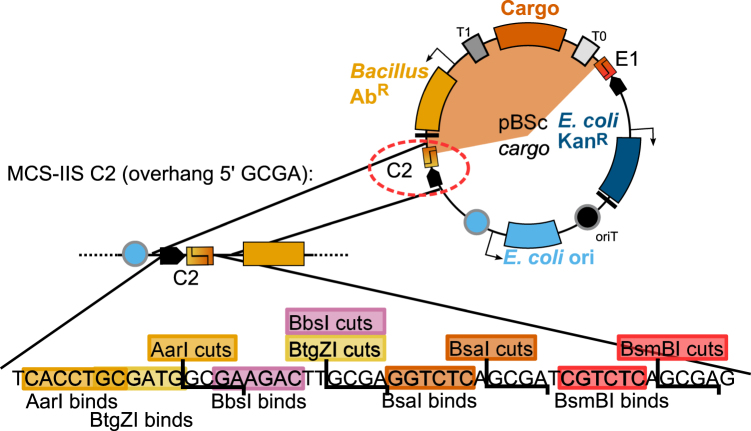
Architecture of the MCS-IIS C2. This DNA-sequence is located on the *cargo vector* between the *E. coli* ori and the *Bacillus* antibiotic marker. The recognition sites for five type IIS restriction enzymes (AarI, BtgZI, BbsI, BsaI, BsmBI), each designed to create a 5′ GCGA-overhang are encoded on the DNA stretch. Architecture of all MCS-IIS can be found in Fig. S1.

All four *entry* parts are flanked by MCSs that contain recognition sites for all five type IIS restriction enzymes (MCS-IIS). Inside each MCS-IIS, recognition and restriction sites are designed so that the same overhang sequence is created, independent of the enzyme used. The overhangs are non-palindromic and differ in at least two nucleotides to ensure the correct assembly of the desired vector. For ease of understanding, fusion sites were named with capital letters B, C, E, and F as indicated in Fig. 2. Figure 3 shows the annotated MCS-IIS C2, in which all five enzymes create the overhang GCGA. For the detailed sequence of all 8 MCS-IIS, see Fig. S1.

To compare the assembly efficiency as a function of the enzyme of choice, we used one set of *entry* vectors to construct *final* vectors with identical features. However, nucleotide sequences differ at the assembly scar sites, due to the MCS-IIS. We used pSEVA23X-amyE (*up*), pSEVA243Y-amyE (*down*), pBSc243M and pBSd141R as *entry* vectors. In this case, the cargo carries a *lacZα*-fragment that allows for blue-white screening and colonies carrying the correct vector appear blue in the presence of 5-bromo-4-chloro-3-indolyl-β-D-galactopyranoside (X-Gal). Red colonies carry the original *destination vector* and white colonies an incorrect vector. We checked colony color, test digest and sequencing results and found good assembly efficiencies for four enzymes: AarI, BbsI, BsaI and BsmBI (Table 2). For BtgzI however, we failed in finding conditions allowing the correct assembly of the *final vector*. This was surprising, since its use in Golden Gate assembly has been described previously in combination with BsmBI^30^. Even if the desired fragments were digested and gel purified separately, the ligation yielded no correct vector. But in principle, BtgZI can be used for assembly once suitable conditions are found. In contrast, BsaI, BpiI and BsmBI were particularly well-suited with efficiencies of >95% in routinely vector assembly (data not shown). The optimized conditions we used for each enzyme are given briefly in the Methods section and are described in more detail in the Supplemental Text S1.

**Table 2:** Assembly efficiencies^*^ depending on restriction enzyme.

Enzyme	% Blue^$^	% White	% Red	Total colonies	Corr. test digest^#^	Corr. Sequencing	*B. subtilis* transf.^§^	
AarI	**36.8 (±26.1)**	0.9 **(±**0.9)	62.2 **(±**25.5)	3017 **(±**1292)	14/18	3/3	++	4/4
BbsI	**41.5 (±34.2)**	0.7 **(±**0.1)	57.7 **(±**34.1)	3568 **(±**1084)	15/18	3/5	++	4/4
BsaI	**52.8 (±13.1)**	6.1 **(±**2.2)	41.1 **(±**13.9)	4004 **(±**1443)	15/18	3/3	++	4/4
BsmBI	**49.8 (±11.0)**	3.7 **(±**0.5)	46.5 **(±**10.9)	2000 **(±**412)	13/18	3/3	++	4/4
BtgZI	**0**	0	100	182	0/6	0/3	n.a.	n.a.

^*^Entry parts: pBSc243M, pBSd141R, pSEVA243X-amyE (*up*) and pSEVA243Y-amyE (*down*).

^$^Colonies appear blue if carrying the correct *final vector* and red if the *destination vector* is unchanged. If available, only blue colonies were used for test digest and correct test digests were used for sequencing. Data is shown for 3 independent Golden Gate assemblies with AarI, BbsI, BsaI or BsmBI. The total number of colonies as well as their apparent color is shown as average and standard deviation. BtgZI did not lead to correct assemblies of *final vector* and data is derived from a single experiment only.

^#^Test digests, sequencing and transformation results are given in total.

^§++^Indicates >1000 colonies per 100 µl of *B. subtilis* W168 transformation mixture. The correct insertion locus was verified with a starch test.

Taken together, *Bacillus* SEVA siblings vectors can be efficiently assembled using one of four type IIS restriction endonucleases. In addition to the established enzymes (BsaI, BbsI and BsmBI), AarI was also found to be suitable for Golden Gate assembly. Because of its 7 bp recognition site, it should be found less frequently in genomic sequences compared to the “classical” enzymes with a 6 bp recognition site.

### Our collection of *entry* parts for the assembly of *Bacillus* SEVA siblings

**Figure 4:**
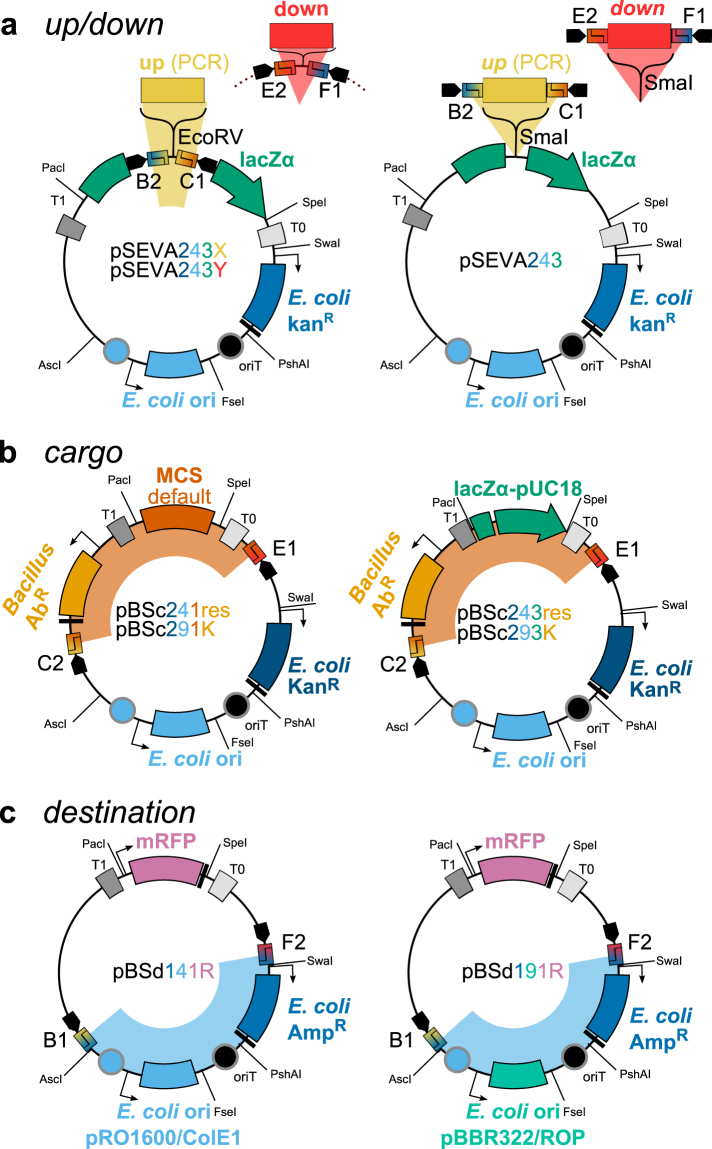
Vector suite for the generation of *Bacillus* SEVA siblings. Schematic representation of the vector architectures, details are listed in Table 1. (**a**) Vectors for flanking homology regions. *Up* fragments (PCR product) can be stored in pSEVA243X and *down* in pSEVA243Y, each linearized with EcoRV. The respective MCS-IIS are encoded on the vectors. If required restriction site are already encoded on the primer overhangs, fragments can be stored in pSEVA243 or used directly for Golden Gate assembly. (**b**) *Cargo vectors* carry one of the following *Bacillus* antibiotic markers: Ble, Cat, Kan, MLS, Spc, Tet, Zeo and either the default MCS (pBSc241res) or the lacZα*-pUC18 MCS for blue/white-screening. Vectors carrying the *Bacillus* kanamycin resistance marker utilize the medium copy number pBR322/ROP ori, all others the high copy number pRO1600/ColE1. Backbones are also available without *Bacillus* marker to allow insertion of a new or customized marker. (**c**) *Destination vectors* carry an mRFP1-cassette as cargo for red/white screening and an ampicillin resistance marker for selection in *E. coli*. They are available with high (pRO1600/ColE1) or medium (pBR322/ROP) copy number origins of replication.

For the assembly of *Bacillus* SEVA siblings vectors, four different categories of *entry* parts are needed: *cargo and resistance*, *up*, *down*, and the *replication part*. They will be described in more detail below. The *entry* vectors offered with our toolbox are depicted in Fig. 4 and listed with detailed descriptions in Table 1.

#### pBSc: Cargo and resistance

Since for most experiments the same cargo will usually be combined with the same resistance marker, both are located on the *cargo vector pBSc*. As a result, only four instead of five parts have to be assembled, thereby increasing cloning efficiency. For our collection of *cargo vectors*, we used well-established antibiotic resistance markers to enable selection on phleomycin D, chloramphenicol, kanamycin, macrolide and streptogramin B antibiotics (MLS), spectinomycin, tetracycline, and zeocin (Table 3). Forbidden restriction sites were removed and transcriptional terminators were added where necessary. Transcription occurs in the opposite direction to the cargo. Per default, the *cargo vector* uses the high copy number ori pRO1600/ColE1. For the kanamycin resistance marker, this vector was unstable, so the medium copy number ori pBR322/ROP was used. All *cargo vectors* are offered with the default SEVA MCS (pUC18-related polylinker without *lacZα*,^7^) or the lacZα*-pUC18 MCS (Table 1). The cargo of choice can be inserted into the *cargo vector* or into the *final vector*, depending on enzyme compatibility and cloning strategy.

**Table 3:** Description of antibiotic markers used in this study.

Abbr.		Gene	Description*	Antibiotic	Conc. [µg ml^−1^]	Source
	amp^r^	*bla*	β-lactamase (Eco)	Ampicillin	100	pSEVA143^7^
	kan^r^	*neo*	neomycin-kanamycin phosphotranferase type I (Eco)	Kanamycin	50	pSEVA241^7^
	cm^r^	*cat*	chloramphenicol *O*-acetyltransferase (Eco)	Chloramphenicol	35^$^	pSEVA341^7^
B	ble^r^	*bleO* ^#^	bleomycin binding protein (phleomycin D)	Phleomycin D1	100	pDG148^40^
C	cm^r^	*cat*	chloramphenicol *O*-acetyltransferase	Chloramphenicol	5	pBS3Clux^17,19^
K	kan^r^	*aph(3’)IIIa*	aminoglycoside *O*-phosphotransferase APH(3’)-IIIa	Kanamycin	10	pDG780^41^
M	MLS^r^	*ermC*	23 S rRNA (adenine(2058)-*N*(6))-methyltransferase	Erythromycin & Lincomycin	1 & 25	pDG647^41^
S	spc^r^	*aad(9*	aminoglycoside nucleotidyltransferase ANT9	Spectinomycin	200	pBS4S^16,17^
T	tet^r^	*tetL*	tetracycline efflux MFS transporter	Tetracycline	12.5	pDG1513^41^
Z	zeo^r^	*ble-Sh* ^#^	phleomycin/bleomycin binding protein (codon-optimized for Bsu)	Zeocin	100	This study

^*^Eco, *E. coli*; Bsu, B. subtilis.

^$^5 µg ml^−1^ were used for medium copy number vectors.

^#^*bleO* and *ble-Sh* both mediate resistance against phleomycin or zeocin (both from the bleomycin family) by binding to the antibiotic, respectively. There are differences in amino acid sequence of the encoded proteins and in the properties of the antibiotics, but mediation of cross-resistance cannot be excluded.

#### Up and down flanking homology regions

The choice of *up* and *down* flanking homology regions depends on and needs to be adjusted to the *Bacillus* strain and experiment. The regions need specific overhangs for the subsequent assembly. This can be achieved by either of the following two strategies: (i) PCR-amplified fragments of choice (without overhangs) are cloned blunt end into the EcoRV-linearized pSEVA243X (*up*) or pSEVA243Y (*down*), respectively. As a result, the insert receives the matching MCS-IIS encoded on these vectors which can be used for Golden Gate assembly. (ii) Incorporation of one restriction site to the primers will directly allow the use of the PCR product for Golden Gate assembly.

#### pBSd: replication part in E. coli

The *destination vector* carries the *replication part* of the *final vector*. Its resistance (ampicillin marker) differs from all other *entry vectors* to allow selection for the correct backbone after vector assembly. A high copy number (pBSd141R) and medium copy number version (pBSd191R) are provided. Both carry an mRFP cassette in their default MCS, thereby allowing red/white screening: After Golden Gate assembly, colonies with the original *destination vector* will appear red and therefore can be discarded. If different *E. coli* features are required for the *final vector*, they can be exchanged in the *destination vector* or the *final vector*, depending on needs and enzyme compatibilities.

### Assembly, transformation and integration of pBS vectors

The reaction for the vector assembly contains the *entry* parts, one of the type IIS restriction enzymes, ligase and buffer. The specific protocols depend on the enzyme used and can be found in the Materials and Method section. Competent *E. coli* cells are subsequently transformed with the reaction mix and plated on selective media containing ampicillin (and X-Gal in case of blue/white screening). Verification of the *final vector* can be achieved in a two-step procedure using test digests followed by sequencing. For the latter, primers TM3782 and TM3783 (if pBSd141R was used as *destination vector*) or TM3783 and TM5128 (pBSd191R) are recommended. The sequencing results should cover the *up* and *down* region as well as the adjacent assembly scars to ensure correct assembly of all parts. We tested all *entry* parts of the toolbox (Table 1) for their assembly efficiencies (Tables S4–S6) and found that in all cases it was more than sufficient to test six colonies of the correct color to obtain a correct *final vector*. If the high copy number *destination vector* is used, or selection for the correct cargo is possible, e.g. via blue/white screening, assembly efficiencies are even higher.

Prior to transformation of *B. subtilis* W168, all vectors have to be linearized, e.g. using ApaI, to ensure chromosomal integration by double homologous recombination. All transformations were successful – except for vectors carrying the tetracycline resistance marker where only for one of two loci could be targeted (Tables S4, S5). The transformation efficiencies depended on the selection marker (Tables 2, 3 and S4–S6). Correct integration was verified by colony PCR or physiological tests; e.g. a starch test for integration into the *amyE* locus.

**Figure 5:**
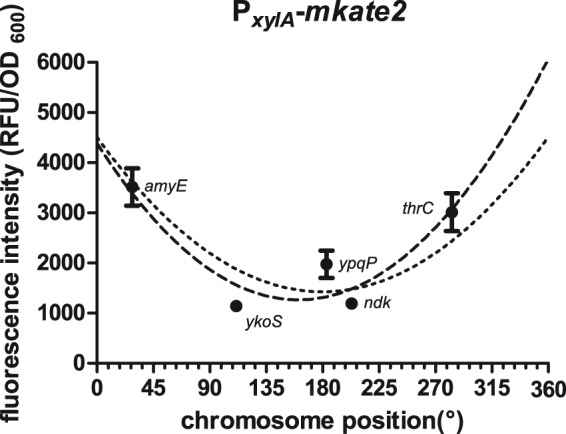
Maximal promoter activity of P_*xylA*_ depends on chromosomal location. The reporter construct P_*xylA*_-*mkate2* was integrated into the *B. subtilis* genome at five different chromosomal loci, as indicated. mKate expression was maximally induced with the addition of 0.2% xylose and fluorescence intensity was measured as an indicator for mKate abundance. The fluorescence intensity is given as a function of the chromosome position. The error bars show the standard deviation of three independent biological replicates. The data was fitted to two second order polynomial functions: dashed line: no constrains, R^2^ = 0.85; dotted line: minimum was set to X = 180, R^2^ = 0.72.

As a proof of concept, we analyzed the expression of the reporter gene *mkate2* under control of the xylose-inducible promoter P_*xylA*_ at five loci spread across the chromosome (Table 4): *amyE*, *ykoS*, *ypqP* (prophage SPβ), *nkd*, and *thrC* at positions 28, 111, 183–195, 203, and 283° on the circular chromosome, respectively. *amyE* and *thrC* are early-discovered and frequently used integration loci close to the ori, encoding a starch-hydrolyzing alpha-amylase and the threonine synthase, respectively. The 130 kb-large prophage SPβ, which itself is inserted at the *ypqP* locus, is known to be not essential and was targeted to demonstrate the possibility of deleting large genomic regions. The two remaining genes, *ndk* and *ykoS*, encode a nucleoside diphosphate kinase and a gene of unknown function, respectively. Those non-essential genes were chosen based on their chromosomal location. Since the same reporter was used in all vector constructs, the reporter-cargo was added at the *entry vector* level to pBSc241M, resulting in pBSc241M-P_*xylA*_-*mkate2*. pBSd141R (ori pRO/ColE1, high copy number) was chosen as the *destination vector* and primers were designed for PCR-product assembly of *up* and *down* fragments via BsaI. Assembly and verification of four *final vectors* was achieved within five working days. The vector carrying homology regions for integration into *ndk* was instable (small colonies), necessitating a change to pBSd191R (ori pBR322/ROP, medium copy number) as a *destination vector*. After transformation of *B. subtilis* W168, the resulting integrants were verified by starch or threonine auxotrophy tests (*amyE* and *thrC*, respectively), or colony PCR for the remaining loci (Table 4). The xylose-inducible promoter P_*xylA*_ was fully induced with xylose in exponentially growing cultures. The fluorescence intensity of *B. subtilis* cells was quantified in triplicates in a microtiter plate reader as a measure for mKate2 production (Fig. 5). The results highlight a dependence of the expression levels on the chromosomal location: As demonstrated previously, genes close to the ori (0°/360°) tend to be expressed at higher levels than those close to the termination region (180°). One exception was the *ykoS*-locus, located at the replication termination site, which had a higher reporter activity than the constructs inserted at the ori-proximal *ypqP* or *ndk* sites. Nevertheless, these results are in good agreement with a recent study^31^, thereby demonstrating that our vector toolbox can for example be used to study the effect of chromosome location on expression levels in a simple and straightforward manner.

**Table 4:** Assembly efficiencies* and reporter activity depending on integration sites.

Locus	**% Light red** ^**$**^	% White	% Red	Total colonies	Correct test digest	Correct Sequencing	*B. subtilis* transf. ^#^	
*amyE*	**69.8**	19.8	10.3	1160	6/6	1/1	+	4/4
*ypqP*	**92.2**	2.9	4.9	1236	6/6	1/1	+	4/4
*ykoS*	**91.6**	3.2	5.3	1900	6/6	1/1	+ +	4/4
*ndk* ^&^		**75**	25	496	12/12	1/1	+ +	4/4
*thrC*	**92.1**	5.2	2.6	4580	6/6	1/1	+	4/4

^*^Data is shown for one Golden Gate assembly using BsaI and the following *entry* parts: PCR products *up* and *down* with BsaI-overhangs, pBSd141R and pBSc141M_P_*xyl*_-*mkate2*.

^$^Colonies appear light red if carrying the correct *final vector* and red if the *destination vector* is unchanged. Only light red colonies were used for test digest and correct test digests were used for sequencing. The correct chromosomal integration was verified as described in Material and Methods.

^#^Number of colonies per 100 µl of transformation mixture: ++, >1000; + , >100 and number of colonies with verified chromosomal integration from number of tested colonies.

^&^pBSd191R was used as *destination vector*. No colonies of “light red” color could be identified due to lower copy number of the *final vector*, in comparison to the other constructs. As a consequence, white colonies were used for further verification.

## Discussion

*B. subtilis* is a versatile heterologous host with powerful genetics allowing precise genomic manipulation. But so far, the available integrative vectors could not easily be adapted to different strains or, more importantly, other *bacilli*, since no modular toolbox was available to allow free combination of resistance markers and integration sites. To fill this gap, we present a rigorously evaluated toolbox for the construction of integrative vectors with customizable flanking homology regions for *Bacillus sp*. The *final* pBS *vector* can be assembled within one week from four *entry* parts via efficient Golden Gate cloning. Our vector suite offers the choice of four type IIS restriction endonucleases (AarI, BbsI, BsaI and BsmBI) for Golden Gate assembly, seven *Bacillus* antibiotic resistance markers, and two different MCSs, available on either a high or medium copy number *E. coli* backbone. The toolbox is adjusted to and widely compatible with the *E. coli* SEVA standard to allow reusability of its cargos or vector parts. All components provided in this toolbox were successfully tested for assembly efficiency, functionality, and usability in *B. subtilis*. The supplementary assembly guide (Text S1) provides necessary information to facilitate a fast and efficient cloning process.

In the course of developing our vectors, a few combinations were discovered which were difficult to handle on high copy number vectors (ori pRO1600/ColE1): (i) The *Bacillus* kanamycin resistance marker was prone to mutations and is therefore offered with the medium copy number ori pBR322/ROP. (ii) The lacZα*-pUC18 MCS differs from the original *lacZα*-pUC18 MCS in a single-nucleotide polymorphism causing a premature stop codon in *lacZα* that does not impair blue/white screening. There was a strong selection pressure against the original *lacZα*-pUC18 MCS (very small colonies), which caused transposon integrations and single nucleotide polymorphisms in *lacZα*. Consequently, we used the mutated but fully functional lacZα*-pUC18 MCS for our constructs. (iii) The *final ndk*-vectors only contained the *ndk down* fragment when using the high copy number pBSd141R as *destination vector*. However, assembly with the medium copy number vector pBSd191R was successful in the first attempt.

Based on our experience, the use of a medium copy number vector is highly recommended as the first trouble shooting strategy in case of cloning issues, especially if slowly growing colonies occur. Also, low copy number oris (e.g. p15A and pSC101) or different directionality of the inserts could be used in case toxicity issues occur, which result in genetic instabilities even on medium copy number vectors.

Furthermore, the tetracycline resistance marker could not confer resistance in *B. subtilis* W168 when the *amyE* locus was targeted. However, when using a different integration locus (*lacA*), the transformation was successful. Due to the dependence of expression levels on the chromosomal region, we suspect this to be causing the transformation issues.

The *Bacillus* SEVA siblings toolbox was developed to provide a versatile starting point for the efficient construction of personalized, yet standardized vectors. Both, the *entry* parts as well as *final vectors* can be customized to meet personal needs. The MCS or cargo and ori can easily be exchanged according to the SEVA standard and new or modified resistance markers can be inserted in markerless *cargo vectors* via AscI and MluI, e.g. markers flanked with target sites for recombinases. These systems would allow directed, recombinase-mediated removal of the marker after chromosomal integration (for reviews on recombinase-mediated cassette exchange, e.g. by Flp and Cre/*loxP*, see^32,33^).

It should also be pointed out that the free choice of chromosomal integration loci provided by our vector toolbox allows for replacing even larger (non-essential) chromosomal areas directly in the process of plasmid integration. We have demonstrated this possibility by integrating an *mkate2*-expressing plasmid into the *ypqP* locus of the *B. subtilis* chromosome, thereby deleting the 130 kb prophage SPβ (Table 4). This combined integration/deletion at any desired chromosomal position will greatly enhance the possibilities in genetically manipulating *B. subtilis*.

The current resistance markers located on *cargo vectors* were tested for their functionality only in *B. subtilis* W168. Since they originate from broad host range vectors, they are expected to be functionally expressed in many low G + C Gram-positive bacteria (phylum *Firmicutes*). The *Bacillus* SEVA siblings might therefore be suitable for e.g. other bacilli, increasing the number of species where customized vectors can be used for genetic manipulation. Indeed, preliminary results from an ongoing study using our vectors reported similar or even better vector assembly efficiencies as those described in Table 2. Choramphenicol- or erythromycin-resistant mutants were readily obtained in *Paenibacillus polymyxa* (order: *Bacillales*, family: *Paenibacillaceae*) (Christoph Engl, personal communication).

If transformation efficiencies are too low, conjugation can be performed using the *oriT* already included in the SEVA standard, which provides efficient transfer into various Gram-negative and -positive hosts^7,34,35^. It can be exchanged with a conjugation marker of choice in the *destination* or *final vector* to meet the needs of the target organism. Recombination efficiency of homologous sequences into the chromosome varies more than 30-fold along the *B. subtilis* chromosome^36^, and even more between species. In general, integration rates can be improved by using longer stretches of DNA or a vector with a temperature-sensitive replicon (e.g. based on pMAD^3,37^). Vector replication not only increases the number of vectors per cell and number of cells carrying a vector (by passing it on to the next generation), but also supports the second recombination event during which the vector backbone is excised from the chromosome^3^. A temperature-sensitive origin of replication can therefore be added to the *destination* or *final vector* to improve recombination with the chromosome.

Moreover, the construction logic described for the *Bacillus* SEVA siblings – that is, the restriction enzymes used for the assembly of the final integrative plasmids – can of course also be applied for developing other SEVA-compatible integration vectors for completely unrelated microorganisms in a similar fashion. But this would require adjusting the corresponding resistance cassettes applicable to these bacteria.

Here, we present the first fully modular, yet standardized vector toolkit for integrative vectors in *B. subtilis* and beyond. We hope that the *Bacillus* SEVA siblings vector toolbox will prove to be useful for projects throughout the *Bacillus* world. In case personalized *entry vectors* (*pBSc*, *pBSd*) are created, we would like to encourage sharing them with the *Bacillus* community, e.g. via the SEVA or BGSC collections, to further improve the tools available for genetically manipulating these powerful organisms.

## Material and Methods

### Bacterial strains and growth conditions

All strains used in this study are listed in Table S1. *B. subtilis* and *E. coli* were routinely grown in lysogeny broth (LB) medium (1% (w/v) tryptone, 0.5% (w/v) yeast extract, 1% (w/v) NaCl) at 37 °C with agitation (220 rpm). Solid media additionally contained 1.5% (w/v) agar. Selective media contained appropriate antibiotics, as provided in Table 3.

### Transformation

*E. coli* (XL1 blue, Agilent Technologies, Santa Clara, CA, USA) competent cells were prepared and transformed according to the rubidium chloride method^38^, achieving ~5*10^6^ colony forming units (CFU) per µg pUC18 DNA. Transformations of *B. subtilis* were carried out as described previously^17,22^. The integration of plasmids into the *B. subtilis* genome was checked on starch plates (*amyE*), with minimal medium lacking threonine (*thrC*) or colony PCR (*lacA*, *ypqP, ykoS, ndk*). Detailed protocols were published previously^17,39^.

### DNA manipulation

Vectors and plasmids used in this study are listed in Table 1. General cloning procedure, such as endonuclease restriction digest, ligation and PCR, was performed with enzymes and buffers from New England Biolabs^®^ (NEB; Ipswich, MA, USA) or Thermo Scientific^TM^ (Waltham, MA, USA) according to the respective protocols. Phusion^®^ polymerase was used for PCRs if the resulting fragment was further used, otherwise OneTaq^®^ was the polymerase of choice. PCR-purification was performed with the *HiYield PCR Gel Extraction/PCR Clean-up Kit* (Süd-Laborbedarf GmbH (SLG), Gauting, Germany). Plasmids were prepared using alkaline lysis and subsequent DNA precipitation. All plasmids created during this study are listed in Table 1, their construction is described in supplemental Table S2 and all primer sequences are given in Table S3.

### Golden Gate assemblies of *final vectors*

Golden Gate assemblies were performed using T4 DNA ligase (30 WU) from Thermo Scientific^TM^ with the accompanied buffer. BsaI, BbsI, BsmBI and BtgZI were purchased from NEB, AarI from Thermo Scientific^TM^. *Entry* parts were diluted to 20 or 40 nM stock concentrations and 2 or 1 µl were used per 15 µl reaction, respectively. For all enzymes, 0.5 µl were used per reaction, except for AarI where 1.5 µl were necessary because of its lower activity. BsaI and AarI are active in ligase buffer, but for AarI the accompanying oligonucleotides were supplemented for optimal efficiency. For BbsI half of the ligase buffer was replaced by NEBuffer 2.1 and for BsmBI half was replaced by NEBuffer 3.1.

The general assembly protocol was 37 °C, 30 min; 16 °C, 30 min; (37 °C, 3 min; 16 °C, 5 min) × 15; 37 °C, 10 min; 50 °C, 10 min; 80 °C, 10 min. The exception was BsmBI, where 55 °C were required for the first incubation step and ligase was only added afterwards. 7.5 µl of the final reaction were used for *E. coli* transformation.

### Measurement of P_*xylA*_ activity

Fluorescence intensity of strains carrying a transcriptional fusion of the xylose-inducible promoter P_*xylA*_ and the fluorescence reporter *mkate2* were assayed using a Synergy^TM^ NEOALPHAB multi-mode microplate reader from BioTek^®^ (Winooski, VT, USA). The reader was controlled using the software Gen5^TM^ (version 2.06). Cells were inoculated 1:1000 from fresh overnight cultures and grown to OD_600_ ~0.2, treated with 0.2% xylose and grown for two hours. Cells were harvested by centrifugation and resuspended in phosphate buffered saline (137 mM NaCl, 2.7 mM KCl, 10 mM Na2HPO4, 1.8 mM KH2PO4, pH 7.4). 200 µl per well in 96-well plates (black walls, clear bottom; Greiner Bio-One, Frickenhausen, Germany) were measured for their OD_600_, and mKate-fluorescence using the monochromator with following parameters: endpoint measurement, gain: 100, excitation wavelength: 588 nm, emission wavelength: 633 nm.

### Data availability

The vectors generated in this study are available from the Bacillus Genetic Stock Center (BGSC, http://www.bgsc.org/, accession numbers ECE701-26) and the SEVA collection (http://wwwuser.cnb.csic.es/~seva/?page_id=19). For sequence information, the following accession numbers apply: GenBank, KY995178 to KY995203; ACS Synthetic Biology Registry (https://acs-registry.jbei.org/, JPUB_008862 to JPUB_008887).

## Electronic supplementary material

Supplementary File 1

Supplementary Information
