# Publisher Correction: *Bacillus* SEVA siblings: A Golden Gate-based toolbox to create personalized integrative vectors for *Bacillus subtilis*

**DOI:** 10.1038/s41598-017-18381-z

**Published:** 2018-01-17

**Authors:** Jara Radeck, Daniel Meyer, Nina Lautenschläger, Thorsten Mascher

**Affiliations:** 1 Institute of Microbiology, Technische Universität (TU) Dresden, 01062 Dresden, Germany; 2 Department of Biology I, Ludwig-Maximilians-Universität (LMU) München, 82152 Planegg-Martinsried, Germany

Correction to: *Scientific Reports* 10.1038/s41598-017-14329-5, published online 26 October 2017

The original version of this Article contained errors.

In the original HTML and PDF versions, there was a typographical error in the Discussion section where:

“The MCS or cargo and ori can easily be exchanged according to the SEVA standard and new or modified resistance markers can be inserted in markerless *cargo vectors* via MluI, e.g. markers flanked with target sites for recombinases”.

now reads:

“The MCS or cargo and ori can easily be exchanged according to the SEVA standard and new or modified resistance markers can be inserted in markerless *cargo vectors* via AscI and MluI, e.g. markers flanked with target sites for recombinases”.

The original PDF version of the Article was incorrectly marked as an uncorrected proof.

In addition, there was a typographical error in the caption of Table 1, where:

“**d**, destination vector/**c**, cargo vector/**f**, final vector”.

now reads:

“**d**, destination vector. **c**, cargo vector. **f**, final vector”.

Finally, a supplementary dataset containing the annotated DNA sequences files of the entry vectors in gbk-format was omitted from the original version of this Article.

These errors have now been corrected in the PDF and HTML versions of the Article.

