# Publisher Correction: *Bacillus* SEVA siblings: A Golden Gate-based toolbox to create personalized integrative vectors for *Bacillus subtilis*

**DOI:** 10.1038/s41598-018-22369-8

**Published:** 2018-03-06

**Authors:** Jara Radeck, Daniel Meyer, Nina Lautenschläger, Thorsten Mascher

**Affiliations:** 1 Institute of Microbiology, Technische Universität (TU) Dresden, 01062 Dresden, Germany; 2 Department of Biology I, Ludwig-Maximilians-Universität (LMU) München, 82152 Planegg-Martinsried, Germany

Correction to: *Scientific Reports* 10.1038/s41598-017-14329-5, published online 26 October 2017

An error occurred after publication resulting in the omission of the Article and Volume numbers from the PDF version of this Article. In addition, the supplemental text was inadvertently removed. These errors have now been corrected in the PDF and HTML versions of this Article.

