# Supplementary material for: Bacillus SEVA siblings: A Golden Gate-based toolbox to create personalized integrative vectors for Bacillus subtilis
Source: Sci Rep. 2017 Oct 26;7:14134. doi: 10.1038/s41598-017-14329-5 (PMC5658365; doi:10.1038/s41598-017-14329-5)
Supplement: Supplementary file 1 [file 41598_2017_14329_MOESM1_ESM.pdf]

Bacillus SEVA siblings: A Golden Gate-based toolbox to create personalized integrative vectors for *Bacillus subtilis*

Jara Radeck, Daniel Meyer, Nina Lautenschläger, Thorsten Mascher (2017)

Supplementary File 1, containing:

Figure S1: Architecture of all MCS-IIS

Table S1. Bacterial strains used in this study

Table S2. Construction of Vectors and Plasmids used in this study

Table S3: Oligonucleotides used in this study

Table S4. Assembly efficiencies for pBSc241-derivatives with pSEVA243XamyE (*up*) , pSEVA243YamyE (*down*), pBSd141R and BsaI.

Table S5. Assembly efficiencies for pBSc243-derivatives with pSEVA243XamyE (*up*) , pSEVA243YamyE (*down*), pBSd141R and BsaI.

Table S6. Efficiencies for BsaI-mediated pBS assembly with mixed combinations of *cargo*, *up*, *down* and *destination* vectors and parts.

Table S7. Legend for Table S6.

Guide: How to build your own *Bacillus* SEVA sibling (pBS) (p. 7-12)

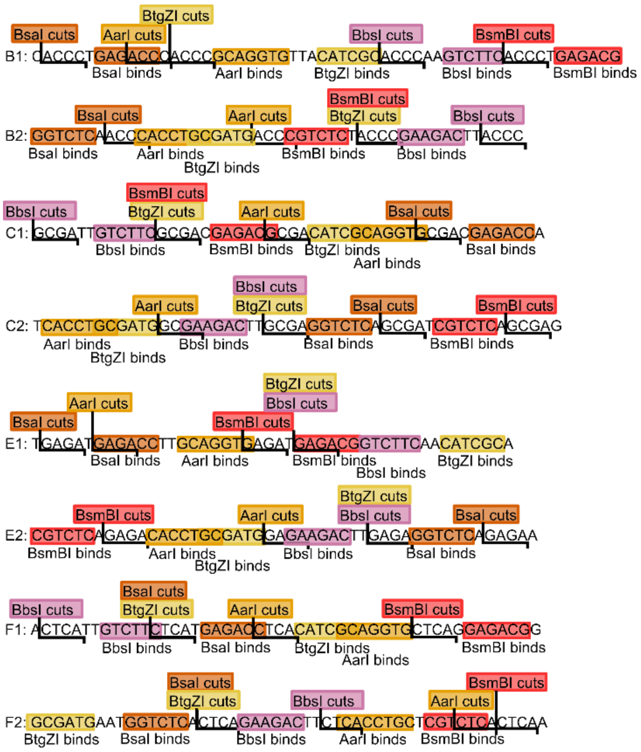

Figure S1: Architecture of all MCS-IIS. These DNA-sequences are located on the *entry vectors* (see Figure 2) and allow the Golden Gate assembly of the *final vector* via one of five type IIS restriction enzymes (AarI, BtgZI, BbsI, BsaI, BsmBI). In each MCS-IIS, the same 4-nt overhang is created, irrespective of the enzyme used. The overhangs are designed to allow assembly in the specified order depicted in Figure 2.

Table S1. Bacterial strains used in this study

Name	Description	Source
E. coli strains		
XL1-Blue	*recA1 endA1 gyrA96 thi-1 hsdR17 supE44 relA1 lac F*'::Tn*10 proAB lacI^q* Δ(*lacZ*)M15]	Agilent Technologies
NEB5α	*fhuA2 Δ(argF-lacZ)U169 phoA glnV44 Φ80 Δ(lacZ)M15 gyrA96 recA1 relA1 endA1 thi-1 hsdR17*	New England Biolabs®
DH5α	*F– Φ80lacZΔM15 Δ(lacZYA-argF) U169 recA1 endA1 hsdR17 (rK–, mK+) phoA supE44 λ– thi-1 gyrA96 relA1*	Thermo Scientific™
B. subtilis strains		
W168	Wild-type, *trpC2*	Laboratory stock
TMB3717	W168 *amyE*::pBSf141mls-amyE_mkate2 (P_{xylA}-*mkate2*, *ermC*)	This study
TMB3718	W168 *ypqP*::pBSf141mls-ypqP_mkate2 (P_{xylA}-*mkate2*, *ermC*)	This study
TMB3719	W168 *ykoS*::pBSf141mls-ykoS_mkate2 (P_{xylA}-*mkate2*, *ermC*)	This study
TMB3720	W168 *ndk*::pBSf191mls-ndk_mkate2 (P_{xylA}-*mkate2*, *ermC*)	This study
TMB3721	W168 *thrC*::pBSf141mls-thrC_mkate2 (P_{xylA}-*mkate2*, *ermC*)	This study

Table S2. Construction of Vectors and Plasmids used in this study

**Name**	**Primers and Enzymes used for cloning**[a]
pBSd141R	800bp DNA-fragment (synthesized by Thermo Scientific™ containing MCS-IIS B1, T1, MCS default, T2, MCS-IIS F2) was ligated into pSEVA243 via SwaI+AscI to create vector pSEVA141BF. Monomeric red fluorescent protein (mRFP BBa_E1010)) was codon-optimized for expression in *Bacillus* with *B. subtilis* W168 *rpsB* promoter and *yvtI* terminator from *Bacillus licheniformis* DSM13, synthesized, cut with KpnI+BbsI and ligated into pSEVA141BF (BamHI+KpnI).
pBSd191R	BsmBI-free pBR322 origin of replication was created by PCR-joining two fragments with primers TM4150+TM4153: TM4150+TM4151, pMAD and TM4152+4153, pMAD. This fragment was cut with FseI+AscI and ligated with the 1500bp and 600bp fragments of pSEVA141BF cut with SwaI, FseI and AscI. mRFP from pBSd141R was inserted via EcoRI+XbaI.
pSEVA243X	DNA-fragment containing MCS-IIS B2, EcoRV and MCS-IIS C1 was synthesized by Thermo Scientific™ and cloned into pSEVA243 via EcoRI+SpeI.
pSEVA243Y	DNA-fragment containing MCS-IIS E2, EcoRV and MCS-IIS F1 was synthesized by Thermo Scientific™ and cloned into pSEVA243 via EcoRI+SpeI.
pBSc241B	*ble*-cassette was amplified by PCR (TM3773+TM3774, pDG148), cut with MluI and ligated into pBSc241 (cut with AscI).
pBSc241C	*cat*-cassette was amplified by PCR (TM3771+TM3772, pBS3Clux), cut with MluI and ligated into pBSc241 (cut AscI).
pBSc241M	SacI-free *mls*-cassette was created by PCR-joining 2 fragments with TM3767+TM3768: TM3767+TM3770, pDG647 and mls-back TM3769+TM3768, pDG647, cut with MluI and ligated into pBSc241 (cut with AscI).
pBSc241S	KpnI+SwaI-free *spec*-cassette (incl. terminator BBa_B0014) was created by PCR-joining 3 fragments with TM3762+TM3761: TM3762+TM3764, pBS4S, TM3763+TM3765, pBS4S and TM3766+TM3761, pSB1A3-mkate-B0014, cut with MluI and ligated into pSEVA241EC (cut with AscI).
pBSc241T	BsmBI+AarI-free *tet*-cassette was created by PCR-joining 2 fragments with TM3752+TM3753: TM3752+TM3755, pDG1513 and TM3754+TM3753, pDG1513, cut with MluI and ligated into pBSc241 (cut with AscI).
pBSc241Z	*zeo*-cassette was synthesized by Thermo Scientific™ (codon adapted for *B. subtilis*, with *kan*-promoter from pDG780 and *cat*-terminator from pBS3Clux flanked with MluI restriction sites), cut with MluI and ligated into pBSc241 (cut with AscI).
pBSc291K	BbsI-free *kan*-cassette (incl. terminator BBa_B0014) was created by PCR-joining of 3 fragments with TM3756+TM3761: TM3756+TM3758, pDG780, TM3757+TM3759, pDG780 and TM3760+TM3761, pSB1A3-mkate-B0014, cut with MluI and ligated into pBSc291 (cut with AscI).
pBSc243B	*lacZα**-pUC18-MCS from pSEVA143* was ligated into pBSc241B via PacI+SpeI.
pBSc243C	*lacZα**-pUC18-MCS from pSEVA143* was ligated into pBSc241C via PacI+SpeI.
pBSc243M	*lacZα**-pUC18-MCS from pSEVA143* was ligated into pBSc241M via PacI+SpeI.
pBSc243S	*lacZα**-pUC18-MCS from pSEVA143* was ligated into pBSc241S via PacI+SpeI.
pBSc243T	*lacZα**-pUC18-MCS from pSEVA143* was ligated into pBSc241T via PacI+SpeI.
pBSc243Z	*lacZα**-pUC18-MCS from pSEVA143* was ligated into pBSc241Z via PacI+SpeI.
pBSc293K	*lacZα**-pUC18-MCS from pBSc243 was ligated into pBSc291K via PacI+SpeI.
pBSc241	DNA-fragment containing MCS-IIS C2, T1, MCS default, T0, MCS-IIS E1 was synthesized by Thermo Scientific™, cut with MluI+ScaI and ligated into pSEVA243 (cut with SwaI+AscI).
pBSc243	*lacZα**-pUC18-MCS from pSEVA143* was ligated into pBSc241 via PacI+SpeI.
pBSc291	BsmBI-free ori pBR322 from pBSd191R cut with AscI+FseI was ligated into pSEVA243 cut with AscI+FseI+SpeI (both 800bp and 1300bp fragments), creating pSEVA293*. DNA-fragment containing MCS-IIS C2, T1, MCS default, T0, MCS-IIS E1 was synthesized by Thermo Scientific™, cut with MluI+ScaI and ligated into pSEVA293* (cut with SwaI+AscI).
pBSc293	*lacZα**-pUC18-MCS from pBSc243 was ligated into pBSc291via PacI+SpeI.
pBSc391	BtgZI/BsmBI/ScaI-free *cat* cassette was synthesized by Thermo Scientific™ and ligated into pSEVA243 via PshAI+SwaI, creating pSEVA343*. BsmBI-free ori pBR322 cut from pBSd191R with AscI+FseI ligated into pSEVA343* cut with AscI+FseI+SpeI (both: 800+1500bp bands were used), creating pSEVA393*. DNA-fragment containing MCS-IIS C2, T1, MCS default, T0, MCS-IIS E1 was synthesized by Thermo Scientific™, cut with MluI+ScaI and ligated into pSEVA393* (SwaI+AscI).
pBSc393	*lacZα**-pUC18-MCS from pBSc243 was ligated into pBSc392 via PacI+SpeI.
pSEVA243X-amyE	"*amyE*-up" fragment (600bp) amplified from W168 with TM4230+TM4231 was ligated into EcoRV-linearized pSEVA243X.
pSEVA243Y-amyE	"*amyE*-do" fragment (600bp) amplified form W168 with TM4981+TM4982 was ligated into EcoRV-linearized pSEVA243Y.
pBSc141M_P_{xylA}-*mkate2*	Insert P_{xylA} (BBa_K1351039, EcoRI+SpeI) and red fluorescent protein *mkate2* (BBa_K823029, codon-adapted to *B. subtilis*, XbaI+PstI) were ligated into pBSc241mls (EcoRI+PstI).
pBS141M-amyE_mkate2	Golden Gate reaction with BsaI and the vectors pBSc141M_P_{xylA}-*mkate2* and pBSd141R, as well as the PCR fragments (~500 bp) BsaI-*amyE*-up (TM4518+TM4519, W168) and BsaI-*amyE*-do (TM4983+TM4984, W168).
pBS141M-ypqP_mkate2	Golden Gate reaction with BsaI and the vectors pBSc141M_P_{xylA}-*mkate2* and pBSd141R, as well as the PCR fragments (~500 bp) BsaI-*ypqP*-up (TM5100+TM5101, W168) and BsaI-*ypqP*-do (TM5102+TM5103, W168).
pBS141M-ykoS_mkate2	Golden Gate reaction with BsaI and the vectors pBSc141M_P_{xylA}-*mkate2* and pBSd141R as well as the PCR fragments (~500 bp) BsaI-*ykoS*-up (TM5108+TM5109, W168) and BsaI-*ykoS*-do (TM5110+TM5111, W168).
pBS191M-ndk_mkate2	Golden Gate reaction with BsaI and the vectors pBSc141M_P_{xylA}-*mkate2* and pBSd191R as well as the PCR fragments (~500 bp) BsaI-*ndk*-up (TM5112+TM5113, W168) and BsaI-*ndk*-do (TM5114+TM5115, W168).
pBS141M-thrC_mkate2	Golden Gate reaction with BsaI and the vectors pBSc141M_P_{xylA}-*mkate2* and pBSd141R as well as the PCR fragments (~500 bp) BsaI-*thrC*-up (TM5116+TM5117, W168) and BsaI-*thrC*-do (TM5118+TM5119, W168).

[a] *lacZα**-pUC18: contains a premature stop codon (C202T => Q68*) in the *lacZα*-open reading frame. *lacZα* is still functional for blue-white screening. Non-mutated fragments (can) lead to plasmid instability if combined with the high copy number ori pRO1600/ColE1.

Table S3: Oligonucleotides used in this study

Number	Name	Sequence[a] (5' to 3')
TM0057	mls-check-fwd	CCTTAAAACATGCAGGAATTGACG
TM0718	cat-check-fwd	AATAGCGACGGAGAGTTAGG
TM0498	kan-check-fwd	GCCGGTATAAAGGGACCACC
TM0058	spec-check-fwd	GTTATCTTGGAGAGAATATTGAATGGAC
TM5222	tet-check-fwd	TGTTTTAGGTGGGCTTTCGTTC
TM3680	pSEVA PS1	AGGGCGGCGGATTTGTCC
TM3681	pSEVA PS2	GCGGCAACCGAGCGTTC
TM3682	pSEVA PS3	GAACGCTCGGTTGCCGC
TM3683	pSEVA PS4	CCAGCCTCGCAGAGCAGG
TM3684	pSEVA PS5	CCCTGCTTCGGGGTCATT
TM3685	pSEVA PS6	GGACAAATCCGCCGCCCT
TM3782	pSEVAcheck-sites1	CGCAAAAAACGCACCACTACG
TM3783	pSEVAcheck-sites2	GGTTATTGTCTCATGAGCGG
TM3784	pSEVAcheck-sites3	CTTGTATTACTGTTTATGTAAGCAG
TM4154	SEVA141BF-T0SpeI-fwd	CTGGCGACTAGTCTTGGAC
TM5128	pSEVAcheck-sites4	GGTTACTGATGATGAACATGC
TM3752	MluI-Tet-fwd	GATC*ACGCGT*GGATTTTATGACCGATGATGAAG
TM3753	MluI-Tet-rev	GATC*ACGCGT*TAAAAAAAGGATCAATTTTGAACTCTC
TM3754	Tet-BsmBI,AarI mut fwd	GAAATGGTTTTGAACGTCagcTTACCTGATATTGCAAATGATTTTAATAAACCtCCTG CGAGTACAAACTGG
TM3755	Tet-BsmBI,AarI mut rev	CCAGTTTGTACTCGCAGGaGGTTTATTAAAATCATTTGCAATATCAGGTAAgctGAC GTTCAAAACCATTTC
TM3756	MluI-kan-fwd	GATC*ACGCGT*TTCTGGTATTTAAGGTTTTAGAATGC
TM3757	Kan-BbsI mut fwd	GGATTGCGAAAACTGGGAAGAgGACACTCCATTTAAAGATCCGC
TM3758	Kan-BbsI mut rev	GCGGATCTTTAAATGGAGTGTCcTCTTCCCAGTTTTCGCAATCC
TM3759	Kan w/o term rev	GAGCCAGTGTGAGGTACTAAAACAATTCATCCAG
TM3760	B0014-kan-fusion fwd	GAATTGTTTTAGTACCTCACACTGGCTCACCTTCG
TM3761	MluI-B0014-rev	GATC*ACGCGT*AAAATAATAAAAAAGCCGGATTAATAATC
TM3762	MluI-spec-fwd	GATC*ACGCGT*TAACAACTATGGATATAAAATAGG
TM3763	Spec-KpnImut fwd	CAATTATTATTCAGCAAGAAATGGTtCCGTGGAATCATCCTCCC
TM3764	Spec-KpnImut rev	GGGAGGATGATTCCACGGaACCATTTCTTGCTGAATAATAATTG
TM3765	Spec-SwaImut-w/oterm rev	TTATAATTTTTTTAATCTGTTgTTTAAATAGTTTATAGTTAAATTTAC
TM3766	B0014-spec-fusion fwd	CTATTTAAAcAACAGATTAAAAAAATTATAATCACACTGGCTCACCTTCG
TM3767	MluI-mls- fwd	GATC*ACGCGT*GATCCTTTAACTCTGGCAACCCTC
TM3768	MluI-mls-rev	GATC*ACGCGT*GCCGACTGCGCAAAAGACATAATC
TM3769	MLS-SacImut fwd	CTCATCATGTTCATATTTATCAGAGgTCGTGCTATAATTATACTAATTTTATAAGG
TM3770	MLS-SacImut rev	CCTTATAAAATTAGTATAATTATAGCACGAcCTCTGATAAATATGAACATGATGAG
TM3771	MluI-cat-fwd	GATC*ACGCGT*AAGTGGGATATTTTTAAAATATATATTTATG
TM3772	MluI-cat-rev	GATC*ACGCGT*CAGGTTAGTGACATTAGAAAACC
TM3773	MluI-bleo-fwd	GATC*ACGCGT*ACGATGACCTCTAATAATTGTTAATC
TM3774	MluI-bleo-rev	GATC*ACGCGT*TCTTTTATTCAGCAATCGCGC
TM4150	pBR322-AscI fwd	TATTTTGGCGCGCCAATATCCCGCCGCATCCATACC
TM4151	pBR322-BsmBImut rev	GCTTACAGACAAGCTGTGACgcTCTCCGGGAGCTGCATG
TM4152	pBR322-BsmBImut fwd	CATGCAGCTCCCGGAGAgcGTCACAGCTTGTCTGTAAGC
TM4153	pBR322-FseI-rev	TTAAATGGCCGGCCCGTAGAAAAGATCAAAGGATC
TM4230	SEVA-amyE-up-fwd	ATGTTTGCAAAACGATTCAAAACC
TM4231	SEVA-amyE-up-rev	CGATCAGACCAGTTTTTAATTTG
TM4981	SEVA-amyE-do fwd	CTGGGCGGTGATAGCTTC
TM4982	SEVA-amyE-do rev	CTTTTGTTGTATTCGCATCTGC
TM4518	BsaI-amyE-up-fwd	CTCAAGG*GGTCTC*C**ACCC**ATGTTTGCAAAACGATTCAAAACC
TM4519	BsaI -amyE-up-rev	CTCAAGG*GGTCTC*C**TCGC**CGATCAGACCAGTTTTTAATTTG
TM4983	BsaI -amyE-do-fwd	CTCAAGG*GGTCTC*C**GAGA**CTGGGCGGTGATAGCTTC
TM4984	BsaI -amyE-do-rev	CTCAAGG*GGTCTC*C**TGAG**CTTTTGTTGTATTCGCATCTGC
TM5100	BsaI-ypqP-up-fwd	CTCAAGG*GGTCTC*C**ACCC**CAAAGTTGAACATATGATGCATGG
TM5101	BsaI-ypqP-up-rev	CTCAAGG*GGTCTC*C**TCGC**GTACTGATATTAATGACATGCTGC
TM5102	BsaI-ypqP-do-fwd	CTCAAGG*GGTCTC*C**GAGA**GTATCTCCTGTGAACACAATGG
TM5103	BsaI-ypqP-do-rev	CTCAAGG*GGTCTC*C**TGAG**GATGCAATTCTTCAATAATCTGAGC
TM5108	BsaI-ykoS-up-fwd	CTCAAGG*GGTCTC*C**ACCC**AAAGGAAGATGCCCATATCCG
TM5109	BsaI-ykoS-up-rev	CTCAAGG*GGTCTC*C**TCGC**CGTCATAAATGCAATACTGCCT
TM5110	BsaI-ykoS-do-fwd	CTCAAGG*GGTCTC*C**GAGA**TTGTTATATACGTGAGCTTTGCG
TM5111	BsaI-ykoS-do-rev	CTCAAGG*GGTCTC*C**TGAG**CGTTTACCGAGTTCATCGAC
TM5112	BsaI-ndk-up-fwd	CTCAAGG*GGTCTC*C**ACCC**CGCAGGAACAGCTTGAACC
TM5113	BsaI-ndk-up-rev	CTCAAGG*GGTCTC*C**TCGC**TGTTCGGCGTAGTGTTTCTC
TM5114	BsaI-ndk-do-fwd	CTCAAGG*GGTCTC*C**GAGA**ACCTGTATTCGCAATGGTGTG
TM5115	BsaI-ndk-do-rev	CTCAAGG*GGTCTC*C**TGAG**AGTCTCGCTGTCTTTTTGTCC
TM5116	BsaI-thrC-up-fwd	CTCAAGG*GGTCTC*C**ACCC**GCGTGAGCTTTGAAAAAATCC
TM5117	BsaI-thrC-up-rev	CTCAAGG*GGTCTC*C**TCGC**AATGCATTTCATGTTAGCACGG
TM5118	BsaI-thrC-do-fwd	CTCAAGG*GGTCTC*C**GAGA**GAAAATCCGGAAACAATAGCG
TM5119	BsaI-thrC-do-rev	CTCAAGG*GGTCTC*C**TGAG**GCTTTCAAAGACGGTCAGC

[a] Endonuclease restriction enzyme recognition sites in italics, restriction site in bold for IIS enzymes; Underlined nucleotides do not anneal with the original template. Introduced mutated nucleotides in lower case.

Tables S4-6. Assembly efficiencies for BSs vectors. Colonies appear blue (Table S4 and first five constructs of Table S6) or white (Table S5 and remaining constructs of Table S6) if carrying the correct *final vector* and red if the *destination vector* is unchanged. Only colonies of the correct color were used for test digest and correct test digests were used for sequencing. Data is shown for one Golden Gate assembly each. Number of colonies per 100 µl of *B. subtilis* W168 transformation mixture: ++, >1000; +, >100; o,>10; θ, <10; -, no colony with verified chromosomal integration. Number of colonies with correct insertion locus as verified with a starch test (out of colonies tested) is given in the last column.

Table S4. Assembly efficiencies for pBSc241-derivatives with pSEVA243XamyE (*up*) , pSEVA243YamyE (*down*), pBSd141R and BsaI.

Cargo vector	% white	% red	Total colonies	Correct test digest	Correct Sequencing	*B. subtilis* transformation	
pBSc241	**90.8**	9.2	8592	3/6	1/1	n.a.	n.a.
pBSc241B	**93.6**	6.4	6336	4/6	1/1	o	2/4
pBSc241C	**86.5**	13.5	4384	3/6	1/2	++	3/4
pBSc291K	**84.5**	15.5	3564	4/6	1/2	o	2/11
pBSc241M	**84.1**	15.9	3328	2/6	1/1	++	3/4
pBSc241S	**90.6**	9.4	6816	5/6	1/1	o	3/10
pBSc241T	**90.3**	9.7	1480	2/6	1/1	-; θ*	0/1;3/3[a]
pBSc241Z	**91.2**	8.8	2448	6/6	1/1	o	4/4

[a] *B. subtilis* transformation was not successful for integration into the *amyE* locus. However, transformation was successful if the *lacA* locus was targeted (data only shown in column "*B. subtilis* transformation").

Table S5. Assembly efficiencies for pBSc243-derivatives with pSEVA243XamyE (*up*) , pSEVA243YamyE (*down*), pBSd141R and BsaI.

Cargo vector	% blue	% white	% red	Total colonies	Correct test digest	Correct Sequencing	*B. subtilis* transformation	
pBSc243	**81.5**	9.6	8.9	7248	6/6	1/1	n.a.	n.a.
pBSc243B	**75.4**	17.8	6.8	4224	6/6	1/1	o	4/4
pBSc243C	**68.0**	18.4	13.6	5056	3/6	1/2	++	4/4
pBSc293K	**76.8**	11.8	11.4	2640	4/6	1/1	o	4/4
pBSc243M	**71.3**	7.2	21.5	6024	3/6	1/1	++	4/4
pBSc243S	**82.8**	4.4	12.8	3248	6/6	1/1	o	7/20
pBSc243T	**78.9**	5.0	16.1	2576	5/6	1/1	-; θ[a]	0/7;9/9[a]
pBSc243Z	**77.9**	16.1	6.0	2988	6/6	1/1	o	4/4

[a] *B. subtilis* transformation was not successful for integration into the *amyE* locus. However, transformation was successful if the *lacA* locus was targeted (data only shown in column "*B. subtilis* transformation").

Table S6. Efficiencies for BsaI-mediated pBS assembly with mixed combinations of *cargo*, *up*, *down* and *destination* vectors and parts.

Abb.[a]	% blue	% white	% red	Total colonies	Correct test digest	Correct Sequencing	*B. subtilis* transformation	
34V	**44.8**	3.0	52.2	3248	6/6	1/1	++	4/4
34P	**73.4**	10.8	15.8	3336	6/6	1/2	+	4/4
39V	**67.7**	19.4	12.9	744	6/6	1/1	++	4/4
39P	**68.0**	30.9	1.1	4200	6/6	1/1	++	4/4
39Pkan	**62.1**	36.6	1.3	3672	6/6	1/1	o	4/4
14V	n.a.	**38.1**	61.9	2560	4/6	1/1	+	4/4
14P	n.a.	**82.0**	18.0	2000	3/6	1/1	++	4/4
19V	n.a.	**95.7**	4.3	1128	3/6	1/1	++	4/4
19P	n.a.	**99.2**	0.8	2380	1/6	1/1	++	4/4
19Pkan	n.a.	**98.6**	1.4	2840	1/6	1/1	o	4/4

[a] The abbreviations encode for *entry* parts and are outlined in Table S7.

Table S7. Legend for Table S6.

Name	Cargo	Destination	Up	Down
34V	pBSc243M	pBSd141R	pSEVA243XamyE	pSEVA243YamyE
34P	pBSc243M	pBSd141R	BsaI-amyE-up (PCR)	BsaI-amyE-down (PCR)
39V	pBSc243M	pBSd191R	pSEVA243XamyE	pSEVA243YamyE
39P	pBSc243M	pBSd191R	BsaI-amyE-up (PCR)	BsaI-amyE-down (PCR)
39Pkan	pBSc293K	pBSd191R	BsaI-amyE-up (PCR)	BsaI-amyE-down (PCR)
14V	pBSc241M	pBSd141R	pSEVA243XamyE	pSEVA243YamyE
14P	pBSc241M	pBSd141R	BsaI-amyE-up (PCR)	BsaI-amyE-down (PCR)
19V	pBSc241M	pBSd191R	pSEVA243XamyE	pSEVA243YamyE
19P	pBSc241M	pBSd191R	BsaI-amyE-up (PCR)	BsaI-amyE-down (PCR)
19Pkan	pBSc291K	pBSd191R	BsaI-amyE-up (PCR)	BsaI-amyE-down (PCR)

How to build your own *Bacillus* SEVA sibling (pBS)

Explanation of *Bacillus* SEVA siblings

1) *Bacillus* SEVA siblings (pBS) are vectors that are made for *Bacillus sp*. and are based on the Standard European Vector Architecture (SEVA) for replicative *E. coli* vectors.

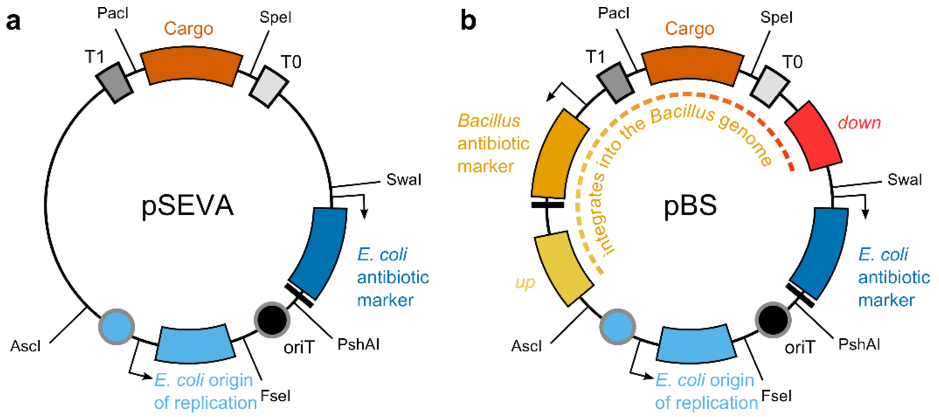

Figure 1. Comparison of SEVA vector (a) and Bacillus SEVA sibling (b)

2) They are assembled from 4 parts (*up*, *cargo-resistance pBSc*, *down*, and *destination pBSd*) via the one-pot Golden Gate assembly (compatible overhangs are marked with the same letter, see Fig. 2), so that the homology regions for genomic integration can be easily adjusted to your strain of interest.
3) Single parts of the vector can be exchanged before or after the assembly according to the SEVA-Standard.
4) List of available *Entry vectors*, see Table 1 and 2: *destination vectors* (*pBSd*); *up* and *down vectors*; *cargo-resistance vectors* (*pBSc*). Sequences can be downloaded from the SEVA and Bacillus Genetic Stock Center (BGSC) collections.

Table 1. Quick guide of *entry* vectors

**Name**	**description**	**Remarks/What to do with it?**
**pSEVA243X**	For *up* fragments	Linearize (EcoRV) and insert your PCR product to receive appropriate MCS-IIS (B2+C1). Allows blue/white screening.
**pSEVA243Y**	For *down* fragments	Linearize (EcoRV) and insert your PCR product to receive appropriate MCS-IIS (E2+F1). Allows blue/white screening.
**pSEVA243[a]**	For *up* and *down* fragments	Linearize (SmaI) and insert PCR product that includes IIS overhangs.
 pBSc241res*<br>**pBSc243res***	***Cargo-resistance vector*** with<br>- default MCS (pUC18)<br>- MCS lacZα-pUC18<br>*Resistances available: -, *ble*, *cat*, *kan*, *mls*, *spec*, *tet*, *zeo*. *kan* is offered in a medium copy vector	Adjust cargo before or after BSs assembly
 pBSd141R<br>**pBSd191R**	***Destination vectors*** with<br>- high copy ori: pRO1600/ColE1<br>- medium copy ori: pBR322/ROP<br>Both carry *mRFP1* for red/white screening	Adjust *E. coli* ori before or after BSs assembly

[a] pSEVA243 seems to be prone to transposal integration in the *lacZα*-region in some *E. coli* strains, as manifesting in white (and only small blue) colonies on X-Gal. In this case, consider changing the strain, ori or lacZα-version.

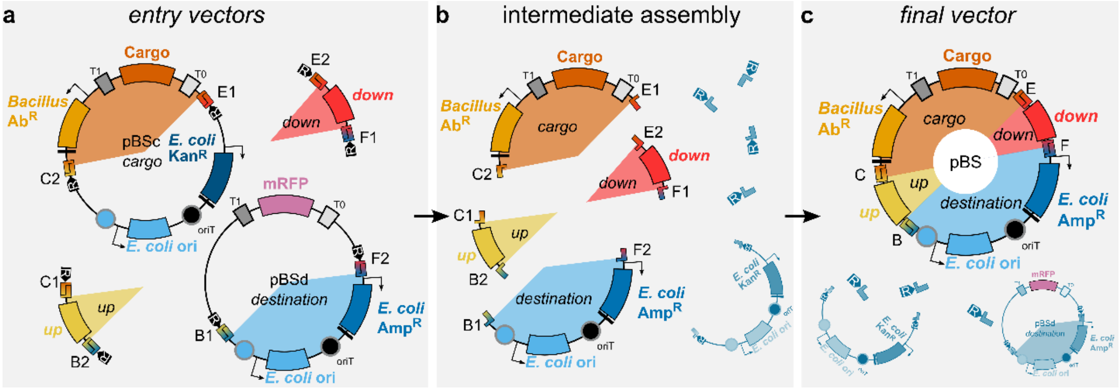

Figure 2. Schematic assembly of *Bacillus* SEVA sibling from *entry* parts

Table 2. Entry vectors available for BSs assembly

**BGSC**[a]	**Name**[b]	**Description**	**Resistance in *E. coli* / *B. subtilis***
**Vectors for default assembly**			
**Destination vectors**			
ECE701	pBSd141R	*mRFP1,* MCS-IIS F2, *bla*, ori pRO1600/ColE1, MCS-IIS B1	Amp^r / -
ECE702	pBSd191R	*mRFP1,* MCS-IIS F2, *bla*, ori pBR322/ROP, MCS-IIS B1	Amp^r / -
**Vectors for flanking homology regions**			
	pSEVA243	*lacZα*-pUC18 MCS, *neo*, ori pRO1600/ColE1	Kan^r/-
ECE703	pSEVA243X	*lacZα**-pUC18 MCS incl. MCS-IIS B2+C1 for *up*, *neo*, ori pRO1600/ColE1	Kan^r/-
ECE704	pSEVA243Y	*lacZα**-pUC18 MCS incl. MCS-IIS E2+F1 for *down*, *neo*, ori pRO1600/ColE1	Kan^r/-
**Cargo-Resistance vectors**			
ECE706	pBSc241B	MCS-default, MCS-IIS E1, *neo*, ori pRO1600/ColE1, MCS-IIS C2, *bleO*	Kan^r/ble^r
ECE707	pBSc241C	MCS-default, MCS-IIS E1, *neo*, ori pRO1600/ColE1, MCS-IIS C2, *cat*	Kan^r/cm^r
ECE708	pBSc241M	MCS-default, MCS-IIS E1, *neo*, ori pRO1600/ColE1, MCS-IIS C2, *ermC*	Kan^r/mls^r
ECE709	pBSc241S	MCS-default, MCS-IIS E1, *neo*, ori pRO1600/ColE1, MCS-IIS C2, *aad(9)*	Kan^r/spc^r
ECE710	pBSc241T	MCS-default, MCS-IIS E1, *neo*, ori pRO1600/ColE1, MCS-IIS C2, *tetL*	Kan^r/tet^r
ECE711	pBSc241Z	MCS-default, MCS-IIS E1, *neo*, ori pRO1600/ColE1, MCS-IIS C2, *ble-Sh*	Kan^r/zeo^r
ECE720	pBSc291K	MCS-default, MCS-IIS E1, *neo*, ori pBR322/ROP, MCS-IIS C2, *aph(3')IIIa*	Kan^r/kan^r
ECE713	pBSc243B	*lacZα**-pUC18 MCS, MCS-IIS E1, *neo*, ori pRO1600/ColE1, MCS-IIS C2, *bleO*	Kan^r/ble^r
ECE714	pBSc243C	*lacZα**-pUC18 MCS, MCS-IIS E1, *neo*, ori pRO1600/ColE1, MCS-IIS C2, *cat*	Kan^r/cm^r
ECE715	pBSc243M	*lacZα**-pUC18 MCS, MCS-IIS E1, *neo*, ori pRO1600/ColE1, MCS-IIS C2, *ermC*	Kan^r/mls^r
ECE716	pBSc243S	*lacZα**-pUC18 MCS, MCS-IIS E1, *neo*, ori pRO1600/ColE1, MCS-IIS C2, aad(9)	Kan^r/spc^r
ECE717	pBSc243T	*lacZα**-pUC18 MCS, MCS-IIS E1, *neo*, ori pRO1600/ColE1, MCS-IIS C2, *tetL*	Kan^r/tet^r
ECE718	pBSc243Z	*lacZα**-pUC18 MCS, MCS-IIS E1, *neo*, ori pRO1600/ColE1, MCS-IIS C2, *ble-Sh*	Kan^r/zeo^r
ECE721	pBSc293K	*lacZα**-pUC18 MCS, MCS-IIS E1, *neo*, ori pBR322/ROP, MCS-IIS C2, *aph(3')IIIa*	Kan^r/kan^r

[a] Bacillus Genetic Stock Center (http://www.bgsc.org/order.php)

[b] Numbers according to SEVA standard: 1st position, resistance marker (1, amp; 2, kan). 2nd position, origin of replication (4, pro1600/colE1 (high copy number). 9, pBR322/ROP (medium copy number). 3rd position, cargo (1, MCS default. 3, lacZα-pUC18 MCS which allows for blue-white screening with X-Gal). For further annotations, please see main publication.

Planning the assembly

Useful questions to plan your vector assembly

1) Is it helpful to insert "your" construct of interest into the cargo region before or after pBS assembly? (test same construct/promoter etc. in different loci or rather use one locus and different cargos?) →both is possible
2) Which resistance marker to choose? (The *tet* marker was not working properly for integration into the *amyE* locus.)

3) Which MCS to choose (with lacZa (= encoded by “3” as a third digit) for blue-white screening, or without (encoded by “1”))?
4) Which locus and homology regions to choose? (For *B. subtilis* >400 bp is recommended; <800 bp is useful for sequencing reasons)
5) Which enzyme (BsaI, BbsI, BsmBI, AarI) to choose for the Golden Gate assembly (check Cargo and homology regions for the respective recognition sites!)? → BsaI is a good default. BtgZI was not working as intended. [Personal experience: the assembly can work fine, even if one unwanted restriction site is present, as long as it is incompatible to the overhangs used for assembly.]
6) Choose destination vector (high (“4” as second digit) or medium copy number(“9”)). For assemblies with parts known to be difficult to handle on high copy number vectors (e.g. the *Bacillus* kan-cassette), the medium copy number version should be chosen.
7) Reusability of integration sites
    a) Use once/few times or only with one of the enzmyes? → use PCR product with overhangs containing the necessary restriction sites. It can also be stored in a vector that does not mediate ampicillin resistance in *E. coli*, e.g. pSEVA243.
    b) Flexibility in choosing the enzyme for the assembly? → use primers without overhangs and insert into pSEVA243X and pSEVA243Y to add overhangs for all enzymes (check for correctly directed insertion!).

Primer design (and cloning) of *up* and *down* integration sites

8) If you choose to only use one enzyme sites (7a): Add the respective overhangs to primers for *up* and *down* integration site (see example primers below) → the fragment can still be stored e.g. in pSEVA243 (via SmaI; direction of ligation into vector is not important) and either the PCR product or the vector can be used for Golden Gate assembly.
9) If you choose to be flexible with the restriction enzyme (7b): Amplify your *up* and *down* fragment with primers without overhang and ligate into EcoRV-site of pSEVA243X (*up*) or pSEVA243Y (*down*). Check for correct direction of integration via sequencing. Use the vector for Golden Gate assembly.
10) If desired, different directionality of the integration part can be achieved by adding overhangs to the *up* and *down* fragments using primer overhangs vice versa to the default version.

Table 2. Example primers with Golden Gate overhangs.

Primer Name	Sequence[a] (5’ to 3’)
BsmBI-sacA-up-fwd	taggct**CGTCTC**t**ACCC**GACAGCACATGACCAGGAG
BsmBI-sacA-up-rev	taggct**CGTCTC**t**TCGC**CCAAAATCGTCCAGCCCG
BsmBI-sacA-do-fwd	taggct**CGTCTC**t**GAGA**CCCATCCGACCATTGACTG
BsmBI-sacA-do-rev	taggct**CGTCTC**t**TGAG**GATTTCCCGGTCGCACTG
BbsI-lacA-up-fwd	taggct**GAAGAC**tt**ACCC**GTGATGTCAAAGCTTGAAAAAAC
BbsI-lacA-up-rev	taggct**GAAGAC**tt**TCGC**ATAAATCACAGTGGCAATCTCC
BbsI-lacA-do-fwd	taggct**GAAGAC**tt**GAGA**TTCAAGCTATATTTGGAGTTGAG
BbsI-lacA-do-rev	taggct**GAAGAC**tt**TGAG**CTAATGTGTGTTTACGACAATTC
BsaI-amyE-up-fwd	ctcaagg**GGTCTC**C**ACCC**ATGTTTGCAAAACGATTCAAAACC
BsaI -amyE-up-rev	ctcaagg**GGTCTC**C**TCGC**CGATCAGACCAGTTTTTAATTTG
BsaI -amyE-do-fwd	ctcaagg**GGTCTC**C**GAGA**ATGGATGAGCGATGATGATATC
BsaI -amyE-do-rev	ctcaagg**GGTCTC**C**TGAG**TCAATGGGGAAGAGAACCG
AarI-amyE-up-fwd	ctcaagg**CACCTGC**aatg**ACCC**ATGTTTGCAAAACGATTCAAAACC
AarI -amyE-up-rev	ctcaagg**CACCTGC**aatg**TCGC**CGATCAGACCAGTTTTTAATTTG
AarI -amyE-do-fwd	ctcaagg**CACCTGC**aatg**GAGA**ATGGATGAGCGATGATGATATC
AarI -amyE-do-rev	ctcaagg**CACCTGC**aatg**TGAG**TCAATGGGGAAGAGAACCG

[a] The **recognition site**, **restriction site** (in all cases a 3' overhang will be generated), and annealing part are indicated. The annealing part needs to specified for "your" fragments. Lower case letters ensure binding of the enzyme to the PCR-product and can be changed, but work fine as they are.

Preparation

11) Make sure that you have all fragments/vectors and the enzymes ready for your assembly. All *entry* vectors except for the destination vector (= *pBSc* and pSEVA243X, pSEVA243Y) mediate kanamycin resistance in *E. coli* (50 µg ml^{-1}), whereas the *destination vectors pBSd* mediate ampicillin resistance (100 µg ml^{-1}).
12) Enzyme supplier: Thermo Scientific™: T4 DNA ligase 30 WU + buffer, AarI + Oligo; New England Biolabs®: BsaI (not "HF"), BsmBI, BbsI, NEBuffer 2.1, 3.1

Assembly

Preparation

1) Prepare and purify vectors or PCR products (Midi, Mini plasmid or PCR prep kits with DNA-binding columns). If you use PCR products, please ensure to clean the product from primer-dimers via gel extraction, since those carry the restriction sites needed for assembly and will be inserted into the *final vector* instead of the correct part.
2) Dilute all vectors and fragments to 40 nM (=fmol/µl), or 20 nM.

Calculation from µg/ml to nM: c (nM) = x (conc. in ng μl^{-1}) *1520/ length (bp)

We recommend estimating the DNA concentration on an agarose gel. For PCR products please ensure there are no "primer clouds" or other fragments as they all contain the correct overhangs and will be part of the assembly!

Golden Gate assembly

For each assembly (in µl)

Cargo (40nM)	1
Destination (40nM)	1
Up (40nM)	1
Down (40nM)	1
T4 DNA-Ligase (30WU)*	0.5
10x BSA	1.5
H_2O	Ad 15⁺

⁺taking into account the enzyme-specific volume given below

Programm:

37°C*	30min	
16°C	30min	X 15
37°C	3min	X 15
16°C	5min	X 15
50°C	10min	
80°C	10min	

Add the following components according to the enzyme to be used:

AarI	1.5	BsaI	0.5	BbsI	0.5	BsmBI*	0.5
50x Oligo	0.3			Puffer 2.1	0.75	Puffer 3.1	0.75
Ligase buffer	1.5	Ligase buffer	1.5	Ligase buffer	0.75	Ligase buffer	0.75

* For BsmBI: initial restriction takes place at 55°C. Add ligase **AFTER** that step.

E. coli transformation into and selection

3) Use competent cells of at least $5*10^6$ CFU/µg DNA. Below 10^6 is not recommended.
4) Plate on selective media (100 µg ml^{-1} ampicillin). For vectors containing *lacZα*, IPTG (1µM) and X-Gal (100 µg ml^{-1}) can be supplemented to allow blue/white-screening.

5) Colonies containing the original destination vector will appear red, those containing the correct vector will be white or blue (if *lacZα* is present). Prolonged incubation or storage can enhance colors of the colonies.
6) Pick colonies for Colony PCR or plasmid preparation (for lacZα also use a replica plate without X-Gal, since "blue" is the "dominant" phenotype over "red") and perform test digest. (e.g. with PvuII or enzymes cutting "your" *up* and *down* fragments or cargo).
7) Verify the correct insert and assembly break points via sequencing (TM3782 and TM3783 for high copy number, TM5128 and TM 3783 for medium copy number vectors).

Table 3. Oligonucleotides recommended for sequencing

Primer Name	Sequence (5' to 3')
TM5128	GGTTACTGATGATGAACATGC
TM3782	CGCAAAAAACGCACCACTACG
TM3783	GGTTATTGTCTCATGAGCGG

Trouble shooting

8) If the ratio of colonies displaying the correct color is far off (20 red: 1 white e.g.), the procedure should be optimized, because most likely those white colonies contain wrong vectors.
9) Correct colonies might be small or not present, if some parts of the vector are toxic in *E. coli*. In this case, the medium copy number destination vector should be chosen.

Naming

The central features of all *entry* and *final vectors* can be described in the SEVA number code. They do not comply with the standard perfectly, so they are called SEVA siblings and will be named:

10) *Entry vectors*: pBSd### for destination vector, pBSc###*res* for cargo vector, pSEVA243X*locus* (=*up*) or pSEVA243-*enzyme*-*locus*-up (if restriction sites were added by primer overhangs), pSEVA243Y*locus* (=*down*) or pSEVA243-*enzyme*-*locus*-down
11) *Final vectors*: pBS###*res*-*locus_cargo (res* = capital letter representing the resistance marker)

Use of the vector

1) Modification of the *final* vector, e.g. by changing the cargo, if applicable.
2) Transformation of your desired organism according to the respective protocols. For *B. subtilis* W168 natural competence can be used (check Radeck *et al*., 2013 for detailed protocols in supplemental material S3)
3) Usually, in the replication part of the vector (=not integrative part), linearization should occur before transformation. Towards that end, e.g. ApaI can be used (check your cargo and integration sites!).
4) Check for spontaneous antibiotic resistant mutants using a "no DNA" control.
5) Verify the correct insertion via Colony PCR of up-fwd Primer / TM3685 (or resistance specific, see below) and TM3682 / down-rev.

Table 4. Oligonucleotides for integration check PCR

Primer Name	Sequence (5' to 3')
TM3682	GAACGCTCGGTTGCCGC
TM3685	GGACAAATCCGCCGCCCT
TM0057 (mls)	CCTTAAAACATGCAGGAATTGACG

TM0718 (cat)	AATAGCGACGGAGAGTTAGG
TM0498 (kan)	GCCGGTATAAAGGGACCACC
TM0058 (spec)	GTTATCTTGGAGAGAATATTGAATGGAC
TM5222 (tet)	TGTTTTAGGTGGGCTTTCGTTC

After performing your experiments & demonstrating functionality:

6) Please submit customized entry vectors to BGSC (http://www.bgsc.org) and/or SEVA (http://wwwuser.cnb.csic.es/~seva/).
